# Efficient Removal of Sulfamethoxazole in Electro-Oxidation System with Boron-Doped Diamond Anode and Electrolyte NaCl: Degradation Mechanisms

**DOI:** 10.3390/molecules30051056

**Published:** 2025-02-25

**Authors:** Xinghui Du, Wenxi Xie, Xianhu Long, Dazhen Li, Weixiong Huang, Igor Ying Zhang, Rongfu Huang

**Affiliations:** 1 Sichuan Provincial Key Laboratory of Universities on Environmental Science and Engineering, MOE Key Laboratory of Deep Earth Science and Engineering, College of Architecture and Environment, Sichuan University, Chengdu 610065, China; 2 MOE Key Laboratory of Groundwater Quality and Health, School of Environmental Studies, China University of Geosciences, Wuhan 430078, China; 3 Shanghai Key Laboratory of Molecular Catalysis and Innovation Materials, Collaborative Innovation Centre of Chemistry for Energy Materials, MOE Laboratory for Computational Physical Science, Shanghai Key Laboratory of Bioactive Small Molecules, Department of Chemistry, Fudan University, Shanghai 200433, China

**Keywords:** electro-oxidation, boron-doped diamond anode, oxidation mechanism, sulfamethoxazole

## Abstract

In recent years, the pollutant sulfamethoxazole (SMX) that is widely used in medical therapy has been frequently detected in different water systems. Thereby, it is necessary to develop green and effective advanced oxidation strategies, especially the electro-oxidation process. In this study, an electro-oxidation system featuring a boron-doped diamond (BDD) anode and NaCl as the supporting electrolyte was implemented to effectively remove sulfamethoxazole (SMX) without the addition of external oxidants. The operational parameters were optimized using the response surface methodology with a pH 7.5, current density of 4.44 mA/cm^2^, and NaCl concentration of 20 mmol/L. The optimization significantly enhanced the degradation efficiency of SMX to obtain 100% removal in 5 min. Results of scavenging and chemical probe experiments indicated the presence of hydroxyl radicals (^•^OH) and chlorine radicals (Cl^•^), with the latter primarily forming between the reaction of Cl^−^ and ^•^OH. A competition experiment further revealed the relative oxidative contribution of Cl^•^ of 38.6%, highlighting its significant role in the degradation process. Additionally, ion chromatography analysis confirmed the presence of Cl^•^ without the formation of harmful by-products such as ClO_4_^−^, affirming the environmentally friendly nature of the system. The toxicity of the degradation by-products was also assessed. The application of current was investigated to explore the influence of coexistence ions as well as repeatability. Overall, this work highlighted the effectiveness of the electro-oxidation system for the degradation of organic pollutants in saline wastewater, demonstrating the significance of optimization of operational parameters for efficient and sustainable environmental remediation.

## 1. Introduction

As a sulfonamide-based antibiotic, the pollutant sulfamethoxazole (SMX) is widely used in animal husbandry; aquaculture; and in the prevention and treatment of diseases caused by microorganisms, e.g., bacterial infections such as urinary tract infections, bronchitis, and prostatitis [1,2,3,4,5,6]. However, SMX has been frequently detected in different environmental media such as sediment, sludge, soil, and wastewater because of the disposal of expired drugs, the incomplete metabolic rate of SMX, and the fact that human excreta still contains large amounts of SMX [3,5,6,7]. SMX has the characteristic of continuous discharge, which can quickly accumulate to higher concentrations [8]. Meanwhile, residual SMX in the environment can be transformed and accumulated in organisms due to its unbiodegradable characteristic, which is likely to lead to the development of drug-resistant genes and drug-resistant bacteria, and thus become potentially harmful to the survival and health of humans and animals [4,5,7]. Therefore, there is a great need for treatment procedures for SMX to eliminate SMX in degraded water environments.

Plenty of advanced water treatment techniques have been developed to remove SMX in wastewater, including adsorption, biological treatment, chemical oxidation, and so on [9,10,11]. The hydrophilic nature of SMX makes activated carbon adsorption less effective [12]. Due to the poor biodegradability of SMX, biological treatment is less efficient in degrading SMX [13]. In contrast, chemical oxidation technology, especially advanced oxidation processes (AOPs), can effectively degrade toxic and refractory organic compounds, providing a powerful way to degrade SMX efficiently [14,15]. In various AOPs, electrochemical oxidation technology has the advantages of no sludge, no chemical consumption, simple operation, safety, and high treatment efficiency [16].

In most of the current research on electro-oxidation systems, Na_2_SO_4_ was used as the electrolyte with additional oxidants [17,18]. However, these studied systems do not accurately fit actual medical wastewater treatment, since actual medical wastewater contains little Na_2_SO_4_. Also, adding external oxidants may cause environmental pollution and increase the economic cost and complexity of the operation. NaCl is a distinct choice as an electrolyte as Cl^−^ is commonly present in wastewater and forms oxidant Cl^•^ radicals on the surface of the Boron-doped diamond (BDD) anode during reactions. The Cl^•^ radical may oxidize organic contaminants [19,20]. It has been demonstrated that reactive chloride species (RCS), such as the strong oxidants Cl^•^ and ClO^•^, with redox potentials (*E*_0_) of 2.0~2.4 V vs. NHE (Normal Hydrogen Electrode) and 1.5~1.8 V vs. NHE, respectively, can effectively oxidize antibiotics through single electron oxidation reactions with electron-rich fractions [21,22,23]. Therefore, this work investigated the electro-oxidative degradation of SMX using NaCl as the electrolyte without adding additional oxidants, which could be more environmentally friendly and easy to operate as well as less economically expensive.

However, the oxidative mechanism for the degradation of SMX in this system is still unclear. It is important to understand the mechanism for developing new electro-oxidation systems. This study established an electro-oxidation system based on a BDD anode and used NaCl as an electrolyte for the degradation of SMX.

In this study, a new electro-oxidation system was developed by using the BDD anode and the electrolyte NaCl, which is often present in saline wastewater, without application of additional oxidants. The operating parameters were firstly optimized to achieve the effective degradation of SMX. The response surface methodology (RSM) was employed to analyze the interactions between different influencing parameters to determine the optimal experimental conditions. The reactive oxygen species and mechanism of the new system were elucidated based on the results of a quenching experiment and ion chromatography experiment. Through an ion chromatography analysis, it was demonstrated that the toxic byproduct ClO_4_^−^ was not produced in the current system. The chemical structure and calculated Fukui index of the SMX molecule were used to identify the degradation byproducts and the degradation pathways. The acute toxicity (LD_50_) of these degradation byproducts was assessed to evaluate the toxicity removal of the system. Finally, the results of the experiments with the addition of anions such as HCO_3_^−^, H_2_PO_4_^−^, SO_4_^2−^, and NO_3_^−^ indicated the potential of the current system to be applied for practical water treatment applications.

## 2. Results and Discussion

### 2.1. Parameter Optimization for Constructing the Electro-Oxidation System

The efficiency of electrochemical degradation of SMX was investigated using a BDD anode and dimensional stable anode (DSA). The results are shown in Figure S1. The rate of SMX degradation using the BDD anode was found to be faster than that of the DSA anode under the same condition, with apparent reaction rate (*k*_obs_) values of 0.97 min^−1^ and 0.96 min^−1^, respectively, indicating the excellent electrochemical activity of the BDD anode. This was due to the higher chlorination and oxygen evolution reaction potentials of the BDD electrode than the DSA electrode [24]. The higher oxygenation potential effectively increases the current efficiency of the electrochemical degradation as well as reducing the production of by-products and energy consumption caused by the easy production of oxygen [24]. Moreover, the BDD electrode exhibits higher electrochemical activity, leading to more ^•^OH and Cl^•^ formation, thus making it more effective in degrading SMX. Therefore, the BDD anode was selected in this work.

**Figure 1:**
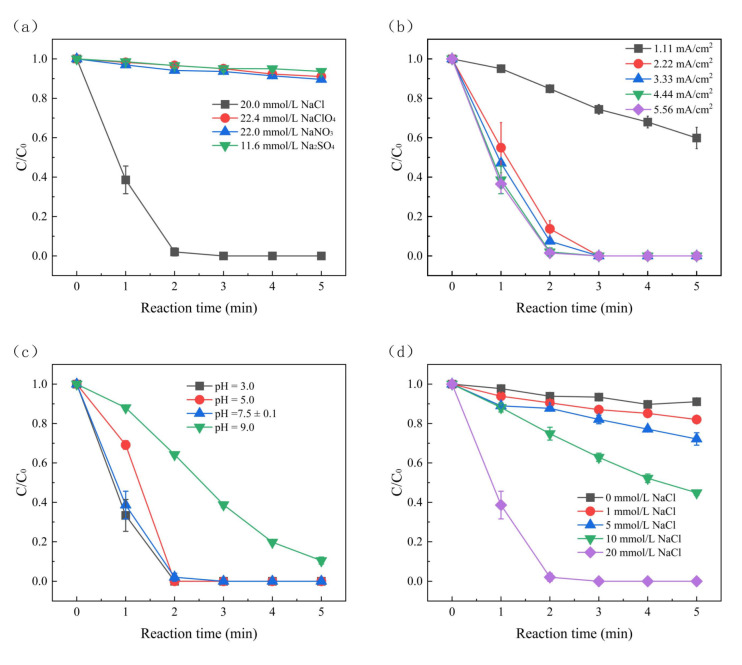
Effect of (**a**) electrolyte, (**b**) current density, (**c**) pH, and (**d**) electrolyte concentration on the SMX degradation in EC/BDD system. Reaction condition: current density = 4.44 mA/cm^2^, [SMX] = 8 μmol/L, pH = 7.5 ± 0.1, conductivity = 2.53 mS, [NaCl] = 20 mmol/L.

The presence of different electrolytes in water can significantly affect the electrolysis process. In this study, the removal rate of SMX using chloride, nitrate, perchlorate, and sodium sulfate as electrolytes was compared. As depicted in Figure 1a, rapid SMX degradation in the NaCl electrolyte was detected, with 100% removal rate (1-C/C_0_) after only 5 min, and the *k*_obs_ achieved 0.97 min^−1^. Similarly, the degradation efficiencies using NaNO_3_, NaClO_4,_ and Na_2_SO_4_ were as follows (in order): NaNO_3_ (10.40%) > NaClO_4_ (8.96%) > Na_2_SO_4_ (6.35%) in 5 min. This is because the abundant Cl^−^ in the system may react with ^•^OH to form additional oxidative species, thus promoting the degradation of SMX [25].

In the electrochemical oxidation process, current density is essential for removing SMX because it determines the transferring rate of the created electrons. As shown in Figure 1b, the removal efficiency of SMX increased from 40.1% to 100%, while the current density increased from 1.11 to 5.56 mA/cm^2^. The corresponding *k*_obs_ also increased from 0.11 to 1.02 min^−1^. This positive effect can be attributed to the higher current density, which accelerated the transfer of electrons through the system and promoted the production of strong oxidants in the system, such as ^•^OH and Cl^•^, thus promoting the removal of SMX [26]. The increased *k*_obs_ was due to the fact that a high current density promotes anodic side reactions such as the oxygen evolution reaction, which reduces the adsorption of pollutant intermediates and maintains the electrode surface’s freshness [27]. Additionally, it is apparent that the removal rate and *k*_obs_ of SMX were similar, with current densities of 4.44 mA/cm^2^ and 5.56 mA/cm^2^. This may be attributed to the low abundance of active species generated in electrolysis. However, when the current density is extraordinarily high, the ions and active species in the solution may not sufficiently diffuse to the electrode surface, resulting in a decrease in the efficiency of the oxidation reaction [28]. According to Equation (1) [25], the energy consumption of the electrolysis process is proportional to the current density, that is, the higher the current density, the higher the energy consumption. With regard to the operational costs and energy consumption, a current density of 4.44 mA/cm^2^ was selected as the optimal condition for practical treatment applications. The influence of different initial pH values (3.0, 5.0, 7.5 ± 0.1, and 9.0) on SMX degradation was investigated. As depicted in Figure 1c, the *k*_obs_ gradually decreased as the pH increased from 7.5 ± 0.1 to 9.0, with a maximum *k*_obs_ of 0.97 min^−1^ at pH 7.5 ± 0.1 and a minimum *k*_obs_ of only 0.47 min^−1^ at pH 9.0. This is because the acidity factors of SMX (pKa_1_ and pKa_2_) were 1.8 and 5.8, respectively. In the case of pH > pKa_2_, the deprotonation of the -NH sulfonamide group enhances the aniline radical oxidation activity [29,30,31]. The *k*_obs_ value was 1.13 min^−1^ at pH 3 but only 0.37 min^−1^ at pH 5. This is because higher oxygen evolution reactions are promoted in acidic conditions, thus inhibiting the production of anodic oxygen and improving current efficiency [32]. The existence of Cl^−^ and ^•^OH radicals may have an effect on the formation of chlorine radicals (Cl^•^) and enhance the degradation of SMX under acidic conditions (Equations (2) and (3)) [33]. Considering the actual operational costs and energy consumption for water treatment, pH 7.5 ± 0.1 was selected as the optimal condition for practical wastewater treatment given that the efficiency of SMX degradation is similar under pH 7.5 ± 0.1 and pH 3. The effect of Cl^−^ (0–20 mmol/L) on the degradation of SMX was also investigated, and the results are shown in Figure 1d. The *k*_obs_ of SMX degradation increased from 0.02 min^−1^ to 0.97 min^−1^, and the Cl^−^ concentration increased from 0 to 20 mmol/L. Further, the SMX degradation rate increased from 8.95% to 100%. Firstly, the conductivity of the solution was increased, and the current efficiency was improved with the increased concentration of NaCl in the system. Secondly, the increase in the chloride ion concentration was a consequence of the subsequent reaction, which accelerated the production of hypochlorite (ClO^−^) and chlorate (ClO_3_^−^), thus indirectly promoting the oxidation rate of the pollutant (Equations (4)–(6)) [34,35,36]. Thirdly, electrolysis oxidizes Cl^−^ to produce free chlorine (i.e., Cl_2_), which could facilitate SMX degradation (Equation (7)) [37].

(1) $$EC_{SMX}=JSV_{C}\Delta t/V_{r}W$$

where EC_SMX_ is the energy consumption (kWh (g SMX)^−1^), J is the current density (A/m^2^), S is the working area of the electrode (m^2^), Vc is the cell potential (V), Δt is the time interval (h), V_r_ is the volume of the electrolyte (m^3^), and W is the SMX decay (mg/m^3^).

(2) $$Cl^{-}{+}^{•}OH\;\to \;ClOH^{•-}$$

(3) $$ClOH^{•-}+H^{+}\to \;Cl^{•}+H_{2}O$$

(4) $$2\;Cl^{-}\to \;Cl_{2}+2e^{-}$$

(5) $$Cl_{2}+H_{2}O\to HOCl+H^{+}+Cl^{-}$$

(6) $$6HOCl+H_{2}O\to \;2Cl{O_{3}}^{-}+14Cl^{-}+12H^{+}+3O_{2}+6e^{-}$$

(7) $$Cl^{-}-e^{-}\to Cl_{2}$$

### 2.2. Optimization of Experimental Conditions by RSM Analysis

**Figure 2:**
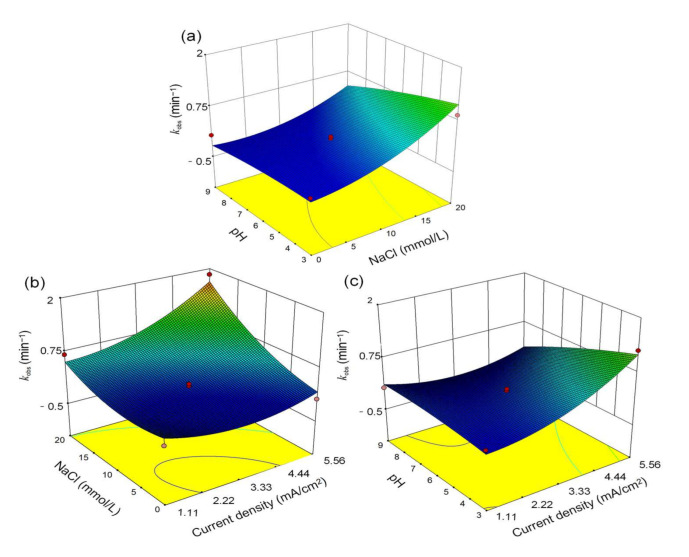
Response surface analysis of the SMX degradation efficiency between (**a**) pH and NaCl concentration, (**b**) current density and NaCl concentration, (**c**) current density and pH. Reaction condition: [SMX] = 8 μmol/L, current density = 1.11–5.56 mA/cm^2^, [NaCl] = 0–20 mmol/L, pH = 3.0–9.0.

The response surface methodology (RSM) was used to explore the synergistic effects of the parameters current density, NaCl concentration, and initial pH to obtain the optimum operating conditions. Considering the synergistic effects of multiple conditions in actual wastewater treatment, a three-factor, three-level design was employed for the experiment, consisting of 17 experimental runs. The statistical method of Analysis of Variance (ANOVA) was applied to analyze the data and interpret the results to test the model’s hypotheses. As shown in Figure 1c, when the pH values were 7.5 ± 0.1 and 3.0, the degradation rate of the system reached 100%; so it cannot be simply assumed that the effect of pH on the degradation rate of the system is monotonous [38,39]. A second-order polynomial model was applied to study the removal rate of SMX as a function of initial pH, NaCl, and current density, according to Equation (8), where A, B, and C represented the current density, NaCl concentration, and initial pH value, respectively [40,41]. A positive term and a negative term in this model were used to indicate the parameter’s facilitative effect on SMX removal and the inhibitory effect, respectively. As shown in Figure 2, the degradation rate of SMX significantly increased when pH < 9 and the NaCl concentration was above 10 mmol/L. This trend was also observed when the current density exceeded 3.33 mA/cm^2^, consistent with the previous results on a single variable. In summary, current density and electrolyte concentration played a more important role than the pH conditions in SMX degradation. According to the model predictions, the optimal conditions for SMX removal were for the conditions of a current density of 5.41 mA/cm^2^, NaCl concentration of 18.18 mmol/L, and pH 3.43, resulting in an SMX degradation *k*_obs_ of 1.90 min^−1^. The model reliability test is shown in Text S2.

(8) $$k_{obs}=+0.13+0.29A+0.45B-0.23C+0.29AB-0.30AC-0.15BC+0.28A^{2}+0.24B^{2}-0.054C^{2}$$

### 2.3. Oxidation Mechanism of SMX in the Electro-Oxidation System

**Figure 3:**
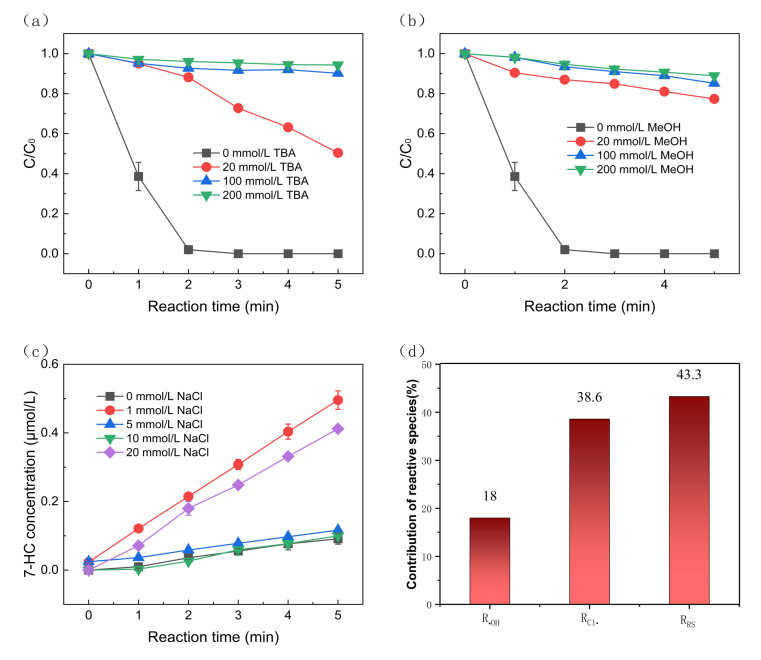
The SMX degradation curves in the electro-oxidation system with the presence of (**a**) TBA, (**b**) methanol. (**c**) Generated 7-HC concentrations in the electro-oxidation system under the condition of different NaCl concentrations. (**d**) Contribution of reactive species to the SMX degradation in the electro-oxidation system. Reaction conditions: current density = 4.44 mA/cm^2^, [SMX] = [BA] = [NB] = 8 μmol/L, pH = 7.5 ± 0.1, [NaCl] = 20 mmol/L.

Scavenging experiments were conducted to evaluate the oxidative species in SMX degradation. TBA (tertiary-butyl alcohol) and methanol were used as the quenching agents [19]. The reaction rates of TBA with ^•^OH and Cl^•^ were 6 × 10^8^ M^−1^ s^−1^ and 3 × 10^8^ M^−1^ s^−1^, respectively [42]. The reaction rate of methanol with ^•^OH was 9.7 × 10^8^ M^−1^ s^−1^. As shown in Figure 3a, the degradation rates of SMX in the electro-oxidation system with the presence of TBA were 49.59%, 9.81%, and 5.66% when TBA concentrations were 20 mmol/L, 100 mmol/L, and 200 mmol/L, respectively. The results indicate the increasing inhibitory effect of TBA on SMX degradation with increasing TBA concentration. Similarly, as shown in Figure 3b, when methanol concentrations were 20 mmol/L, 100 mmol/L, and 200 mmol/L in the electro-oxidation system, SMX degradation rates after 5 min were 22.65%, 14.82%, and 11.05%, respectively, indicating that the inhibition of SMX degradation becomes more prominent with an increase in methanol concentration than the system in the presence of TBA. Through the above two radical quenching experiments, it can be inferred that ^•^OH and Cl^•^ are present in the reaction system, which determines the degradation of SMX.

The effect of NaCl concentration on the production of ^•^OH radicals was also investigated. The ^•^OH in this experiment was derived from water oxidation in the anode since no oxidizing agents were added [43]. Coumarin was used as the ^•^OH probe. It can react with ^•^OH to form 7-hydroxycoumarin [44]. As shown in Figure 3c, the ^•^OH yield decreased from 0.50 μmol/L to 0.10 μmol/L after 5 min, while the NaCl concentration increased from 1 mmol/L to 10 mmol/L. However, the change in ^•^OH yield was more affected as NaCl concentration increased from 0 to 1 mmol/L and from 10 mmol/L to 20 mmol/L. This phenomenon could be explained by the fact that NaCl can both promote the production of ^•^OH and react with ^•^OH, and both competing directions exist simultaneously. When the former reaction dominates, ^•^OH production will increase, and vice versa. The low yield of ^•^OH in the system is because no additional oxidant was added. The presence of ^•^OH and Cl^−^ in the system was demonstrated. Based on Equations (1) and (2), it can be concluded that Cl^•^ is also produced in the system.

Kinetic competition experiments were carried out to quantify the contribution of each reactive substance to pollutant degradation in the electro-oxidation system. These experiments aimed to obtain the contribution of ^•^OH, Cl^•^, and other reactive substances as well as non-radicals to the experimental SMX removal. The steady-state concentrations were used to evaluate the number of reactive substances formed in the AOPs using the BA, NB, and SMX coexistence systems in the competition experiments. NB is the ^•^OH probe and can react rapidly with ^•^OH (3.2 × 10^9^ M^−1^ s^−1^) while having a negligible reaction with Cl^•^ [45]. BA can react rapidly with both ^•^OH and Cl^•^, with secondary rate constants of 4.3 × 10^9^ M^−1^ s^−1^. Both •OH and Cl• can oxidize SMX. The rate constant between ^•^OH and SMX was 6.78 × 10^9^ M^−1^ s^−1^, and the rate constant between Cl^•^ and SMX was estimated as 7.46 × 10^9^ M^−1^ s^−1^ [45]. Figure S2 shows the SMX, BA, and NB degradation in the competitive system, which reflects the apparent reaction rate constants for removing each substance in the competitive experiment. Steady-state concentrations of ^•^OH and Cl^•^ were determined using NB and BA. NB is the typical probe of •OH. As shown in Text S3, the steady-state concentrations of ^•^OH and Cl^•^ were 2.61 × 10^−13^ M and 5.1 × 10^−13^ M, respectively, which were much higher than the yields of free radicals in other oxidation systems [45]. This indicates the practical degradation ability of the experimental degradation system.

**Figure 4:**
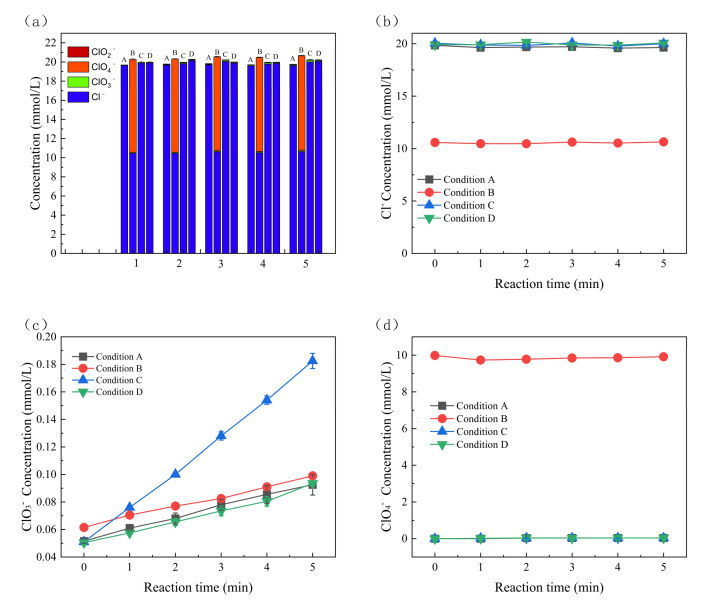
Ion chromatography experiments. Concentration of (**a**) Cl^−^, ClO_3_^−^, ClO_4_^−^, ClO_2_^−^, (**b**) Cl^−^, (**c**) ClO_3_^−^, (**d**) ClO_4_^−^. Reaction conditions: current density = 4.44 or 11.1 mA/cm^2^, [SMX] = 8 or 0 μmol/L, pH = 7.5 ± 0.1, [NaCl] = 20 or 10 mmol/L. NaClO_4_ was used as electrolyte supplement when NaCl concentration was adjusted. Condition A: 4.44 mA/cm^2^ (current density) + 20 mmol/L (NaCl concentration) + 8 μmol/L (SMX concentration); condition B: 4.44 mA/cm^2^ (current density) + 10 mmol/L (NaCl concentration) + 8 μmol/L (SMX concentration); condition C: 11.11 mA/cm^2^ (current density) + 20 mmol/L (NaCl concentration) + 8 μmol/L (SMX concentration); condition D: 4.44 mA/cm^2^ (current density) + 20 mmol/L (NaCl concentration).

Figure 3d and Text S3 show the relative contributions of ^•^OH and Cl^•^ to SMX, respectively. The degradation contributions were 17.98% and 38.58% by ^•^OH and Cl^•^, respectively. The general contribution of other reactive substances and non-radicals (e.g., direct electron transfer (DET) on the anode) to SMX degradation was 43.44%. This indicates that Cl^•^ plays a significant role in the degradation of SMX in the electro-oxidation system. The content of Cl^−^, ClO_2_^−^, ClO_3_^−^, and ClO_4_^−^ was analyzed by ion chromatography to examine their changes under different reaction conditions during the reaction process. The detection of ClO_2_^−^ and ClO_4_^−^ was mainly investigated to examine whether the system produced additional byproducts that were harmful to the environment. As illustrated in Figure 4a, the total sum of elemental chlorine remained relatively constant, which suggests that the BDD electrode and graphite electrode used in the experiment had minimal adsorption effects, thereby powerfully demonstrating the significant degradation effect of the present degradation system. As shown in Figure 4b–d, the concentrations of Cl^−^ and ClO_4_^−^ remained relatively stable over time during the reaction, and the concentration of ClO_4_^−^ in the system was almost zero. This is because the formation of ClO_4_^−^ usually requires a higher electric potential, with sufficient oxygen or excess chlorate in the system [46]. In this reaction system, Cl^−^ was not excessively converted to ClO_4_^−^, which avoided the impact of the degradation process on the environment.

However, ClO_3_^−^ increased significantly under all conditions, further indicating the presence of Cl^•^ in the system, as described by Equations (9)–(11). The change in ClO_3_^−^ yield was small when the NaCl and SMX concentrations were varied, indicating that the conditions of NaCl and SMX concentration had no significant effect on the Cl^•^. When the current density was increased from 4.45 mA/cm^2^ to 11.11 mA/cm^2^, the ClO_3_^−^ yield increased from 0.09 mmol/L to 0.18 mmol/L after 5 min, indicating a significant effect of current on the production of ClO_3_^−^. Alternatively, ClO_2_^−^ was not detected in the system because it is a reactive intermediate with low abundance (Equation (12)) [47].

(9) $$Cl^{•}+Cl^{-}\to C{l_{2}}^{•-}$$

(10) $$C{l_{2}}^{•-}{+}^{•}OH\to HClO+Cl^{-}$$

(11) $$6HOCl+H_{2}O\to \;2Cl{O_{3}}^{-}+14Cl^{-}+12H^{+}+3O_{2}+6e^{-}$$

(12) $$ClO^{-}{+}^{•}OH⟶Cl{O_{2}}^{-}{+}^{•}OH⟶ClO_{3}$$

### 2.4. Byproducts of SMX Degradation Identification and Toxicity Evaluation

Table S2 lists the 25 by-products resulting from the degradation of SMX in the system. Figures S3–S36 shows the mass spectra of each product and its fragment ions. The active sites on the molecules were evaluated using Fukui index calculations [48,49,50]. The results of the calculations are shown in Figure 5b, with C1, C3, C4, C5, and C6 being the main targets of attack. Combining these results with Figure 5a, it can be seen that the most reactive sites in SMX are all located on the benzene ring. This may be because the highest occupied molecular orbital (HOMO) of the SMX molecule is predominantly on one side of the benzene ring [51,52].

**Figure 5:**
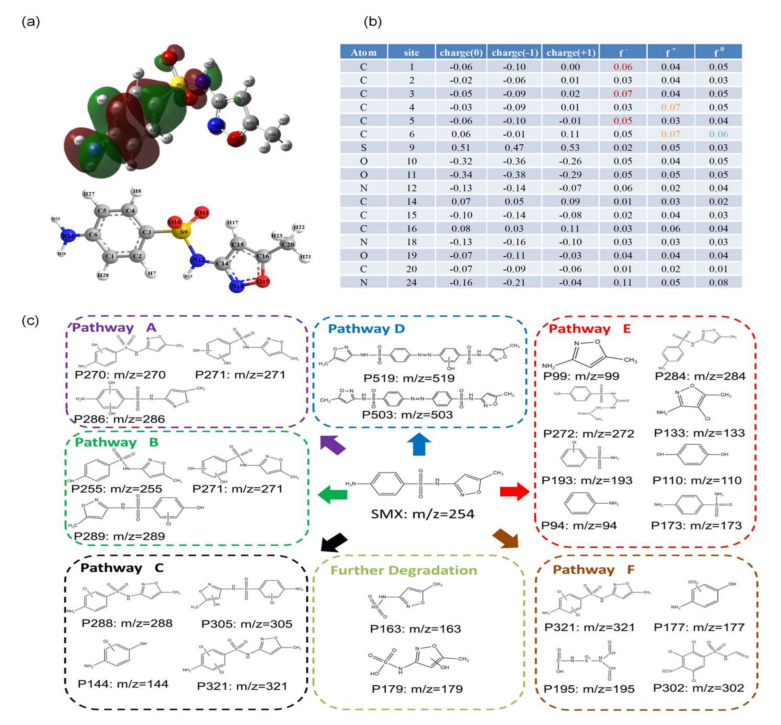
(**a**) Chemical structure of SMX. (**b**) Fukui index of SMX. (**c**) Degradation pathways of SMX in the electro-oxidation systems. Reaction condition: current density = 4.44 mA/cm^2^, [SMX] = 8 μmol/L, [NaCl] = 20 mmol/L, pH = 7.5 ± 0.1. The SMX degradation byproducts were identified using ultra-high-performance liquid chromatography–quadrupole time-of-flight mass spectrometry (UHPLC-QTOF-MS, Agilent 6545, Santa Clara, CA, USA).

The transformation pathways during the oxidation of SMX in the built electro-oxidation system were investigated and summarized in six ways. As shown in Figure 5c, SMX is hydroxylated to produce P270, which is further hydroxylated and chlorinated to produce P271, P286, and P321 in path A. In path B, SMX is oxidized to produce P255, which is further hydroxylated, oxidized, and chlorinated to produce P271, P284, and P289. In pathway C, SMX generates P305, P288, and P144 through hydroxylation, oxidation, and bond breakage. In path D, the SMX generates P519 and P503 by coupling. In path E, SMX generates P272 and P302 by bond breakage. Further small molecule products converted in the degradation pathway include P99, P177, P195, P133, P193, P110, P94, P173, P163, P179. From the above analysis, it is clear that there are two main pathways for the degradation of SMX: conversion (e.g., hydroxylation, chlorination) and bond breakage.

To investigate the various environmental effects of SMX and its degradation by-products, four evaluation indexes, including acute toxicity (LD_50_), bioaccumulation factors, mutagenicity, and developmental toxicity of SMX and its by-products through toxicity estimation software (T.E.S.T. v4.2.1) and quantitative constitutive relationship prediction (QSAR, Figs) models were conducted. Table S3 and Figure S37 show these by-products’ calculated data of biotoxicity. As shown in Figure S37a, the LD_50_ values of SMX and about 85% of its intermediates were more significant than 500 mg/kg, and they can be considered “less toxic”. Thus, the acute toxicity of the system tended to decrease after the degradation of pollutants in this electro-oxidation system. As shown in Figure S37b, the bioaccumulation factors of this system decreased after electrolysis treatment. Many by-products can be degraded to P163 and then to P179, which has a lower bioaccumulation factor of 0.71 than SMX, indicating that this degradation system can effectively reduce the bioaccumulation factor of pollutants. Figure S37c illustrates the developmental toxicant of SMX and its by-products. Up to nine intermediates with less developmental toxicity in this degradation system show the effectiveness of reducing the toxicity of the by-products. In addition, Figure S37d shows the mutagenicity values of the by-products of SMX degradation. The overall negative mutagenicity of the SMX system after electrocatalytic oxidation indicates that the environmental impact of this degradation method is low. The toxicity calculations demonstrate that although some of the by-products produced during the degradation process are more toxic, their transformation pathway indicates that they can be eliminated in the subsequent treatment process. Therefore, it can be concluded that the degradation system can effectively reduce the overall toxicity of SMX.

### 2.5. Practical Wastewater Treatment by EC/BDD/NaCl

**Figure 6:**
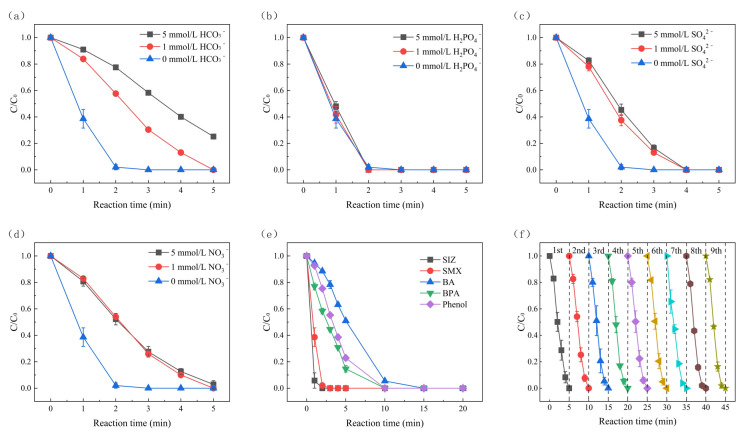
Effects of (**a**) HCO_3_^−^, (**b**) H_2_PO_4_^−^, (**c**) SO_4_^2−^, and (**d**) NO_3_^−^ on the degradation of SMX in the electro-oxidation system. (**e**) Degradation experiments of different pollutants. (**f**) Lifetime experiments. Reaction conditions: current density = 4.44 mA/cm^2^, [SMX] = [SIZ] = [BA] = [BPA] = [Phenol] = 8 μmol/L, [NaCl] = 20 mmol/L, pH = 7.5 ± 0.1.

Figure 6a–d shows the SMX degradation results with the addition of low concentrations of HCO_3_^−^, H_2_PO_4_^−^, SO_4_^2−^, and NO_3_^−^, respectively. All of them had little effect on the degradation of SMX in the built electro-oxidation system, indicating that this system has strong potential for practical application. This is because their anions can react with ^•^OH [53,54], which could also produce other free radicals such as sulfate radicals [55] and H_2_PO_4_^−^ radicals [56].

The experimental system was used to explore the degradation effect of five pollutants. As shown in Figure 6e, the experimental system effectively achieved > 90% degradation rate of SMX, SIZ, BPA, phenol, and BA. Specifically, BPA, SIZ, and SMX reached 80% degradation within 5 min and complete degradation of all five pollutants at 15 min. Notably, the system showed exceptional degradation efficiency for SIZ and SMX, achieving nearly 100% degradation for both pollutants within 2 min. This suggests that the electrolytic sodium chloride system may have a unique affinity for the molecular structure of sulfonamides, enabling rapid degradation without the addition of oxidants. These results demonstrate that this degradation system has great potential for effectively treating pollutants in wastewater. Figure 6f explores the stability and reliability of the degradation of pollutants in the electrolytic sodium chloride system. Under the same reaction conditions, the same set of BDD and graphite electrodes were used to degrade SMX for nine consecutive reactions, and the degradation of SMX in each reaction was recorded. It can be seen that the degradation efficiency of SMX remained at 100% without cleaning the BDD and graphite electrodes, and the degradation rates of all nine consecutive reactions were roughly the same. Therefore, it can be concluded that the degradation system is reliable even after repeated use, with excellent stability and reusability.

## 3. Materials and Methods

### 3.1. Chemicals

Sulfamethoxazole (SMX), sulfisoxazole (SIZ), bisphenol A (BPA), benzoic acid (BA), nitrobenzene (NB), tert-butanol (TBA), sodium nitrate (NaNO_3_), anhydrous sodium dihydrogen phosphate (NaH_2_PO_4_), sodium bicarbonate (NaHCO_3_), sodium chloride (NaCl), sodium perchlorate (NaClO_4_), and coumarin were purchased from Adamas-beta^®^ Company of China (Shanghai, China). Sulfuric acid (H_2_SO_4_), anhydrous sodium sulfate (Na_2_SO_4_), and sodium stearate (NaOH) were obtained from Chengdu Kelong Chemical Reagent Factory (Chengdu, China). All chemicals were used as received without further purification, and all solutions were prepared with deionized water (>18.2 MΩ cm^−1^; MilliQ system, Merck Millipore, Burlington, MA, USA).

### 3.2. Degradation Experiment

The electrochemical catalytic oxidation device was a cylindrical quartz glass reactor with a diameter of 6.5 cm and a height of 9.5 cm. The BDD anode (3 cm × 3 cm × 0.1 cm) and graphite cathode (3 cm × 3 cm × 0.1 cm) were placed in the reactor at a distance of 18 mm with a magnetic bar stirring continuously for mass transfer. The power supply was a DC power supply (APS3003S-3D, GRATTEN, Nanjing, China), and the current density was maintained at 4.45 mA/cm^2^ during the experiment based on optimization results. Each 150 mL solution was used for the degradation with an initial SMX concentration of 8 μmol/L and NaCl concentration of 20 mmol/L as the supporting electrolyte. Each 1 mL sample was taken at specific reaction time points and then injected into HPLC vials prefilled with 20 μL of Na_2_S_2_O_3_ to quench oxidative species in the sample prior to instrumental analysis. The electrodes and reactors were cleaned with ultrapure water and sonicated after the degradation experiment for 1 h to remove residual compounds adsorbed on the surfaces of the electrodes and reactors. All experiments were conducted with a control sample, and all data in the plots were average values of duplicate samples with calculated error bars.

### 3.3. Analytical Method of Pollutants and Byproducts

The concentrations of SMX, benzoic acid (BA), nitrobenzene (NB), sulfisoxazole (SIZ), phenol, and bisphenol A (BPA) were determined using high-performance liquid chromatography (HPLC, 1260 Infinity, Agilent, Santa Clara, CA, USA) with an Eclipse XDB C-18 column (5 μm, 250 × 4.6 mm, Agilent, Santa Clara, CA, USA) and an ultraviolet detector. Other parameters are shown in Table S1. Ultra-high-performance liquid chromatography–quadrupole time-of-flight mass spectrometry (UHPLC-QTOF-MS, Agilent 6545, Santa Clara, CA, USA) was used to characterize the degradation byproducts of SMX. Experimental details are shown in Text S1, and experimental results are shown in Table S1.

### 3.4. Other Analytical Methods

The ion chromatography model was a Thermo Scientific^TM^ Dionex^TM^ Aquion^TM^ RFIC^TM^ (Thermo Fisher Scientific, Waltham, MA, USA) with a Dionex IonPac AS11-HC (4 × 250 mm) column, 25 mmol/L KOH as the eluent, and a flow rate of 1.5 mL/min.

^•^OH was determined using coumarin as a chemical probe, and a concentration of 7-hydroxycoumarin (7-HC) was analyzed using high-performance liquid chromatography (HPLC) equipped with a fluorescence detector (Shimadzu’s remarkable RF-20A, Kyoto, Japan) with a mobile phase composition of 0.1% formic acid in methanol. The flow rate was 1 mL/min, and the column temperature was 30 °C. Fluorescence intensity was measured using an FLR detector with an excitation wavelength of 346 nm and a detection wavelength of 456 nm.

### 3.5. Theoretical Calculation

Density functional (DFT) calculations were performed using Gaussian 09 package and were geometrically optimized using B3LYP, which in turn investigated the molecular structure of the contaminants. Solvent effects of water were considered using a density-based solvation model (SMD). Fukui indices included nucleophilic (*f*^+^), electrophilic (*f*^−^), and radical attacks (*f*^0^), defined respectively as:

Nucleophilic reaction:

(13) $$f_{A}^{+}=q_{N}^{A}-q_{N+1}^{A}$$

Electrophilic reaction:

(14) $$f_{A}^{-}=q_{N-1}^{A}-q_{N}^{A}$$

Radical reaction:

(15) $$f_{A}^{0}=(q_{N-1}^{A}-q_{N+1}^{A})/2$$

where q^A^ is the atomic charge in atom A in the corresponding state. In this work, the free radicals were the main reactive species in the process, and therefore, the Fukui indices *f*^−^ and *f*^0^ were used to analyze the degradation route of SMX [57,58,59].

## 4. Conclusions

In this work, the novel electro-oxidation system reached 100% SMX degradation in 5 min, indicating excellent oxidative capability. The operational conditions were optimized at 20 mmol/L of NaCl concentration, 4.44 mA/cm^2^ of current density, and 7.5 of pH using RSM. The primary oxidative species were Cl^•^ and ^•^OH, and degradation contributions were 17.98% and 38.58%. Cl^•^ was produced via the reaction of Cl^−^ and ^•^OH with no other toxic substances. The degradation mechanisms of SMX were hydroxylation, chlorination, deamination, desulfurization, and bond breaking. As an electrolyte, NaCl produced potent oxidative species that were absent in NaNO_3_, Na_2_SO_4_, and NaClO_4_. Overall, the current BDD/NaCl system achieved effective degradation of SMX with no addition of electrolyte and pH adjustment and satisfactory reusability, making the system advantageous in the treatment of organic pollutants in saline wastewater.

## Data Availability

Data will be provided upon request.

## Supplementary Materials

The following supporting information can be downloaded at: https://www.mdpi.com/article/10.3390/molecules30051056/s1, Figure S1: Comparison of the efficiency of BDD and DSA electrodes for the electrochemical degradation of SMX; Figure S2: The degradation of SMX, BA, and NB in the competitive system; Figure S3: Identification of SMX (*m*/*z* +254) and its fragment ions 156 and 99 ([M + H]^+^); Figure S4: Identification of P284 (*m*/*z* +284) and its fragment ions 84 and 163 ([M + H]^+^); Figure S5: Identification of P284 (*m*/*z* +284) and its fragment ions 99 ([M + H]^+^); Figure S6: Identification of P284 (*m*/*z* +284) and its fragment ions 203 ([M + H]^+^); Figure S7: Identification of P271 (*m*/*z* +271) and its fragment ions 162 ([M + H]^+^); Figure S8: Identification of P271 (*m*/*z* +271) and its fragment ions 174 ([M + H]^+^); Figure S9: Identification of P270 (*m*/*z* +270) and its fragment ions 157 ([M + H]^+^); Figure S10: Identification of P255 (*m*/*z* +255) and its fragment ions 158, 173, and 162 ([M + H]^+^); Figure S11: Identification of P179 (*m*/*z* +179) and its fragment ions 97, 84, and 115 ([M + H]^+^); Figure S12: Identification of P99 (*m*/*z* +99) and its fragment ions 85 and 72 ([M + H]^+^); Figure S13: Identification of P163 (*m*/*z* +163) and its fragment ions 84 ([M + H]^+^); Figure S14: Identification of P163 (*m*/*z* +163) and its fragment ions 149 ([M + H]^+^); Figure S15: Identification of P321 (*m*/*z* +321) and its fragment ions 84 ([M + H]^+^); Figure S16: Identification of P321 (*m*/*z* +321) and its fragment ions 190 ([M + H]^+^); Figure S17: Identification of P302 (*m*/*z* +302) and its fragment ions 259 and 194 ([M + H]^+^); Figure S18: Identification of P288 (*m*/*z* +288) and its fragment ions 84 and 206 ([M + H]^+^); Figure S19: Identification of P177 (*m*/*z* +177) and its fragment ions 143 and 161 ([M + H]^+^); Figure S20: Identification of P144 (*m*/*z* +144) and its fragment ions 128 ([M + H]^+^); Figure S21: Identification of P133 (*m*/*z* +133) and its fragment ions 85 ([M + H]^+^); Figure S22: Identification of P133 (*m*/*z* +133) and its fragment ions 119 ([M + H]^+^); Figure S23: Identification of P286 (*m*/*z* +286) and its fragment ions 124 and 205 ([M + H]^+^); Figure S24: Identification of P272 (*m*/*z* +272) and its fragment ions 90 and 156 ([M + H]^+^); Figure S25: Identification of P503 (*m*/*z* +503) and its fragment ions 238 and 267 ([M + H]^+^); Figure S26: Identification of P519 (*m*/*z* +519) and its fragment ions 283 and 327 ([M + H]^+^); Figure S27: Identification of P194 (*m*/*z* +194) and its fragment ions 176.9 ([M + H]^+^); Figure S28: Identification of P94 (*m*/*z* +94) and its fragment ions 78 ([M + H]^+^); Figure S29: Identification of P173 (*m*/*z* +173) and its fragment ions 81 and 94 ([M + H]^+^); Figure S30: Identification of P289 (*m*/*z* +289) and its fragment ions 191 ([M + H]^+^); Figure S31: Identification of P289 (*m*/*z* +289) and its fragment ions 162 and 207 ([M + H]^+^); Figure S32: Identification of P305 (*m*/*z* +305) and its fragment ions 179 ([M + H]^+^); Figure S33: Identification of P305 (*m*/*z* +305) and its fragment ions 206 ([M + H]^+^); Figure S34: Identification of P195 (*m*/*z* +195) and its fragment ions 124 and 73 ([M + H]^+^); Figure S35: Identification of P195 (*m*/*z* +195) and its fragment ions 136 ([M + H]^+^); Figure S36: Identification of P111 (*m*/*z* +111) and its fragment ions 95 ([M + H]^+^); Figure S37: (a) Acute toxicity, (b) bioaccumulation factor, (c) developmental toxicity and (d) mutagenicity; Table S1: HPLC parameters for detecting different compounds; Table S2: Identified degradation byproducts of SMX by UHPLC-QTOF-MS analysis; Table S3: Identified degradation byproducts toxicity of SMX by toxicity test analysis; Text S1: UHPLC-QTOF-MS identification of degradation byproducts of SMX; Text S2: Model reliability test; Text S3: Probe method to determine steady state concentration of radicals.
