# Supplementary material for: Efficient Removal of Sulfamethoxazole in Electro-Oxidation System with Boron-Doped Diamond Anode and Electrolyte NaCl: Degradation Mechanisms
Source: Molecules. 2025 Feb 25;30(5):1056. doi: 10.3390/molecules30051056 (PMC11901543; doi:10.3390/molecules30051056)
Supplement: Supplementary file 1 [file molecules-30-01056-s001.zip › molecules-3452903-supplementary.pdf]

Supplementary Materials

Efficient Removal of Sulfamethoxazole in Electro-Oxidation System with Boron-Doped Diamond Anode and Electrolyte NaCl: Degradation Mechanisms

Xinghui Du [1,†], Wenxi Xie [1,†], Xianhu Long [1], Dazhen Li [1], Weixiong Huang [2,*], Igor Ying Zhang [3] and Rongfu Huang [1,*]

[1] Sichuan Provincial Key Laboratory of Universities on Environmental Science and Engineering, MOE Key Laboratory of Deep Earth Science and Engineering, College of Architecture and Environment, Sichuan University, Chengdu 610065, China; 2020141470094@stu.scu.edu.cn (X.D.); 2022141470361@stu.scu.edu.cn (W.X.); longxh5@mail2.sysu.edu.cn (X.L.); lidazhen2001@stu.scu.edu.cn (D.L.)

[2] MOE Key Laboratory of Groundwater Quality and Health, School of Environmental Studies, China
University of Geosciences, Wuhan 430078, China

[3] Shanghai Key Laboratory of Molecular Catalysis and Innovation Materials, Collaborative Innovation Centre of Chemistry for Energy Materials, MOE Laboratory for Computational Physical Science, Shanghai Key Laboratory of Bioactive Small Molecules, Department of Chemistry, Fudan University, Shanghai 200433, China; igor_zhangying@fudan.edu.cn

* Correspondence: huangwx@cug.edu.cn (W.H.); rongfu@scu.edu.cn (R.H.); Tel./Fax: +86-191-8176-6064 (R.H.)

† These authors contributed equally to this work.

Total page: 25

Number of texts: 8

Number of tables: 3

Number of figures: 37

Text S1. UHPLC-QTOF-MS identification of degradation byproducts of SMX

The degradation byproducts of SMX during NaCl electrolysis were identified by ultra-high performance liquid chromatography quadrupole time-of-flight mass spectrometry (UHPLC-QTOF-MS) and electrospray ionization sources. BEH C18 column (100 mm × 2.1 mm, 1.7 μm; Waters, Milford, USA) at a column temperature of 45 ° C. Mobile phase A is water containing formic acid (0.1%, v/v) and mobile phase B is 100% methanol. In terms of elution gradient, mobile phase B was set at 5% for the first 3 minutes, increased from 5% to 50% in 7 minutes, then increased from 50% to 95% in 8 minutes and remained there for 4 minutes, and finally mobile phase B was reduced from 95% to 5% in 1 minute and remained there for 2 minutes. The MS and MS/MS modes of SMX and its degradation byproducts were analyzed in positive and negative spray mode, with m/z ranges of 50 to 1000. Based on the accurate m/z values and isotopic patterns, the molecular structure of the identified products was proposed.

Text S2. Model reliability test

The F-value of 10.67 for this experimental model indicates that the model is significant, and there is only a 0.25% chance that such a large F-value could be due to noise. A "Prob > F" value of less than 0.05 indicates that the model term is significant. In this case, A, B, C, AB, AC, A^2, and B^2 are significant model terms. The "lack of fit F-value" for this experimental model is 87.89, meaning that the lack of fit is significant, and there is only a 0.04% chance that such a large "lack of fit F-value" is due to noise. The "Adeq accuracy" measures the ratio of signal to noise, and a ratio greater than 4 is ideal. The ratio for this experimental model was 11.97, indicating that there was sufficient signal. In conclusion, the model fitted for this experiment is reliable, and the results obtained using this model to explore the synergy of the factors and to predict the best response conditions are reasonable.

Text S3. Probe method to determine steady state concentration of radicals

Steady-state concentrations of $^{\bullet}OH$ and $Cl^{\bullet}$ were determined using nitrobenzene and benzoic acid. Nitrobenzene is the typical probe of $^{\bullet}OH$. First, the experimentally observed quasi-level decay rate of nitrobenzene (k_{obs}) was obtained and $[^{\bullet}OH]_{ss}$ was calculated according to Eq. S1.

$$-\ln(\frac{[NB]_t}{[NB]_0}) = k_{HO-NB}\left[{}^{\bullet}OH\right]_{ss} t \quad \text{(Eq.S1)}$$

$[NB]_t$ is the concentration of nitrobenzene at moment t; $[NB]_0$ is the initial concentration of nitrobenzene; $k_{HO\text{-}NB}$ is the primary rate constant of $^{\bullet}OH$ and nitrobenzene, i.e. 3.2×10^9 $M^{-1}s^{-1}$.

$[Cl^{\bullet}]_{ss}$ is calculated using the following Eq. S2.

$$-\ln(\frac{[BA]_t}{[BA]_0}) = (k_{HO-BA}\left[{}^{\bullet}OH\right]_{ss} + k_{Cl-BA}\left[Cl^{\bullet}\right]_{ss})t \quad \text{(Eq.S2)}$$

$k_{HO\text{-}BA}=4.3\times10^9$ $M^{-1}s^{-1}$; $k_{Cl\text{-}BA}=1.8\times10^{10}$ $M^{-1}s^{-1}$

The contribution of each free radical to the oxidation of SMX is calculated by Eqs. S3–S5.

$$R_{\cdot OH} = \frac{k_{OH-SMX}\left[{}^{\bullet}OH\right]_{ss}}{k_{obs,SMX}} \quad \text{(Eq.S3)}$$

$$R_{Cl^{\bullet}} = \frac{k_{Cl-SMX}\left[Cl^{\bullet}\right]_{ss}}{k_{obs,SMX}} \quad \text{(Eq.S4)}$$

$$R_{RS} = 1 - R_{\cdot OH} - R_{Cl^{\bullet}} \quad \text{(Eq.S5)}$$

$k_{HO\text{-}SMX}=6.78 \times 10^9$ M^{-1} s^{-1}; $k_{Cl\text{-}SMX}=7.46 \times 10^9$ M^{-1} s^{-1}

Table S1. HPLC parameters for detecting different compounds.

Compound	Wavelength (nm)	Mobile phase A		Mobile phase B	
		Ultrapure Water (%)	0.1% (v/v) acetic acid solution (%)	Acetonitrile (%)	Methanol (%)
SMX	265	N.A.	60	40	N.A.
BPA	230	30	N.A.	N.A.	70
BA	230	50	N.A.	50	N.A.
NB	262	N.A.	50	50	N.A.
SIZ	271	N.A.	60	40	N.A.
Phenol	274	N.A.	40	60	N.A.

Table S2. Identified degradation byproducts of SMX by UHPLC-QTOF-MS analysis.

Product	ESI (+/-)	Rt (min)	Calculated m/z	Observed m/z	Molecular formula	Proposed structure
SMX	+	9.519	254.0594	254.0596	$C_{10}H_{11}O_3N_3S$	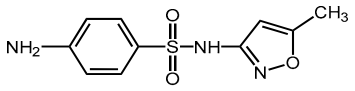
P270[1]	+	14.123	270.0543	270.0543	$C_{10}H_{11}O_4N_3S$	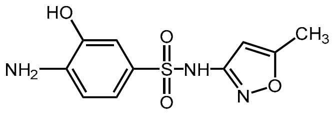
P270[2]	+	14.123	270.0543	270.0543	$C_{10}H_{11}O_4N_3S$	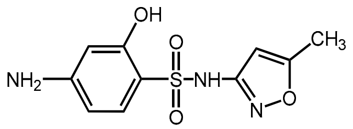
P270[3]	+	9.233	270.0543	270.0543	$C_{10}H_{11}O_4N_3S$	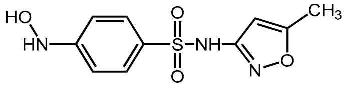
P284	+	8.625	284.0336	284.0336	$C_{10}H_9O_5N_3S$	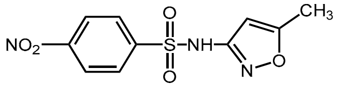
P255	+	10.686	255.0434	255.0438	$C_{10}H_{10}O_4N_2S$	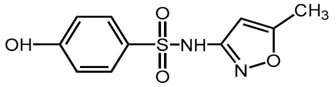
P271[1]	+	9.788	271.0383	271.0394	$C_{10}H_{10}O_5N_2S$	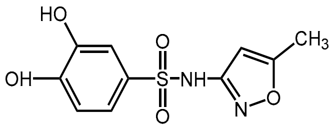
P271[2]	+	9.788	271.0383	271.0394	$C_{10}H_{10}O_5N_2S$	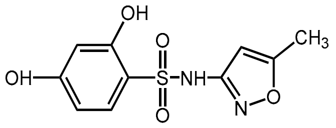
P286	+	9.609	286.0349	286.0500	$C_{10}H_{11}O_5N_3S$	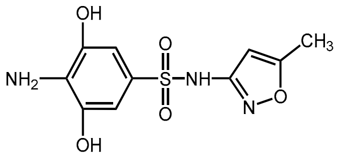
P272	+	9.507	272.0700	272.0630	$C_{10}H_{13}O_4N_3S$	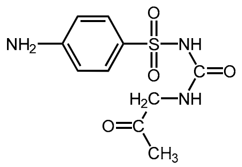
P503	+	14.971	503.0802	503.0808	$C_{20}H_{18}O_6N_6S_2$	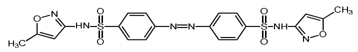

P519[1]	+	21.640	519.0751	519.0751	$C_{20}H_{18}O_7N_6S_2$	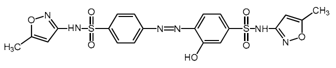
P519[2]	+	21.640	519.0751	519.0751	$C_{20}H_{18}O_7N_6S_2$	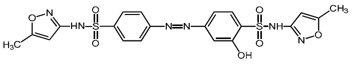
P163	+	8.934	163.9769	163.0190	$C_4H_6O_3N_2S$	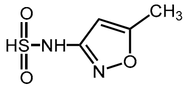
P179	+	5.486	179.0121	179.0123	$C_4H_6O_4N_2S$	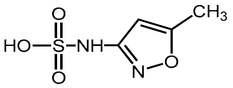
P99	+	3.678	99.0553	99.0553	$C_4H_6ON_2$	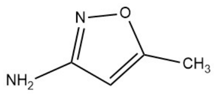
P321[1]	+	13.169	321.9814	321.9818	$C_{10}H_9O_3N_3SCl_2$	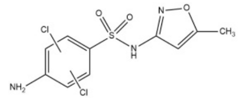
P321[2]	+	8.613	321.9814	321.9828	$C_{10}H_9O_3N_3SCl_2$	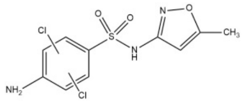
P302	+	24.547	302.9159	302.9188	$C_7H_5O_3N_2SCl_3$	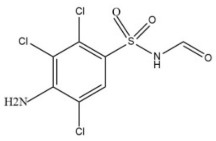
P288	+	11.865	288.0204	288.0209	$C_{10}H_{10}O_3N_3SCl$	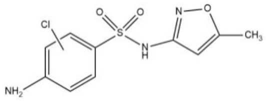
P177	+	11.271	177.9821	177.9823	$C_6H_5ONCl_2$	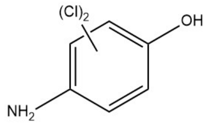
P144	+	2.724	144.0211	144.0225	C_6H_6ONCl	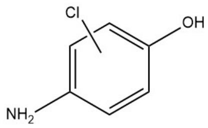
P133	+	5.066	133.0163	133.0155	$C_4H_5ON_2Cl$	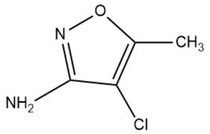
P193	+	6.000	194.0043	194.0043	$C_6H_8O_2NSCl$	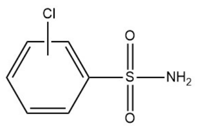

P94	+	15.22	94.0648	94.0648	C_6H_7N	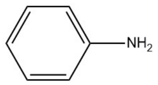
P173	+	7.855	173.0395	173.0395	$C_6H_8O_2N_2S$	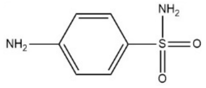
P289	+	12.290	289.0062	289.0062	$C_{10}H_9O_4N_2SCl$	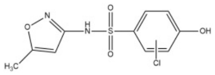
P305	+	12.110	305.0250	305.0250	$C_{10}H_{11}O_4N_3SCl$	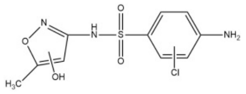
P195	+	24.470	195.9925	195.9925	$C_4H_5O_6NS$	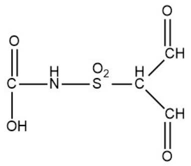
P110	+	18.080	111.0434	111.0434	$C_6H_6O_2$	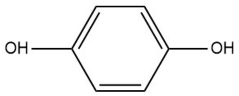

Table S3. Identified degradation byproducts toxicity of SMX by toxicity test analysis.

Product	Oral rat LD$_{50}$ (mg/kg)	Bioaccumulation factor	Developmental Toxicity	Mutagenicity	Proposed structure
SMX	7089.36	2.09	0.76	-0.08	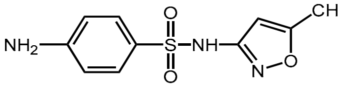
P270^1	2322.28	3.68	0.79	-0.27	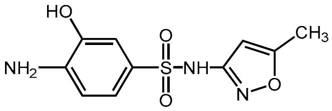
P270^2	7414.18	5.11	0.91	-0.11	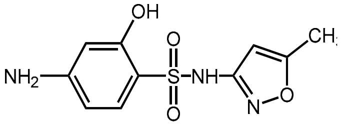
P270^3	2755.55	2.48	0.84	-0.35	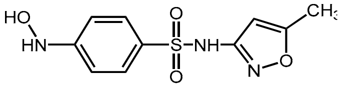
P284	3153.85	2.43	0.83	0	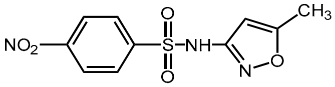
P255	8344.68	3.72	0.82	-0.14	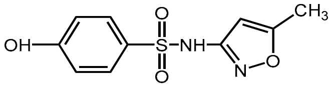
P271^1	2594.68	2.1	0.77	-0.34	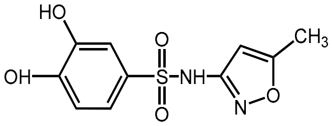
P271^2	7746.37	3.61	0.88	-0.18	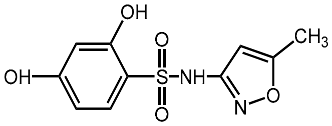
P286	1267.3	3.71	0.74	-0.41	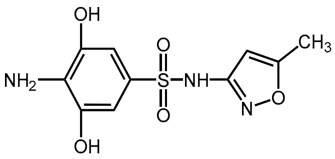

P272	N/A	0.77	0.96	-0.01	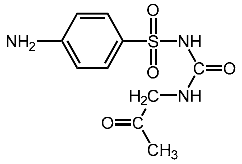
P503	620.36	2.11	0.73	-0.19	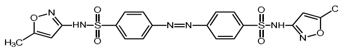
P519[1]	753.57	1.03	0.87	0.14	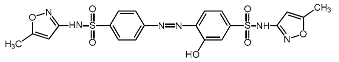
P519[2]	824.82	2.06	0.9	0.01	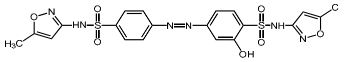
P163	N/A	N/A	N/A	N/A	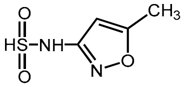
P179	2978.89	0.71	1	0.05	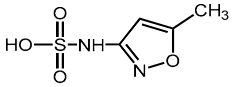
P99	383.09	1.51	0.3	0.44	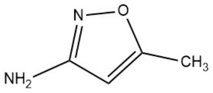
P321[1]	2443.57	10.88	0.85	-0.22	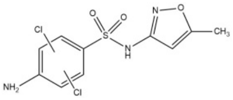
P321[2]	5661.71	10.81	0.9	-0.11	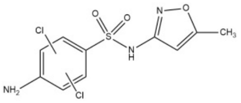
P302	2362.01	11.48	1.18	-0.11	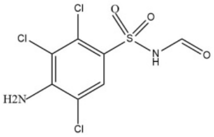
P288	6748.48	4.35	0.84	-0.07	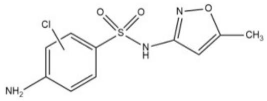
P177	809.49	37	0.58	0.05	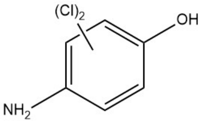

P144	480.88	5.33	0.58	0.05	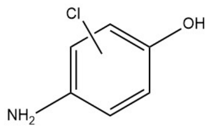
P133	290.78	4.76	0.56	0.46	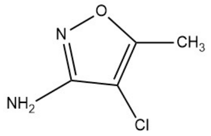
P193	3005.69	2.81	0.44	-0.35	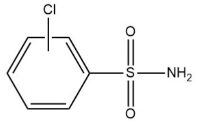
P94	299.91	2.71	0.4	0.38	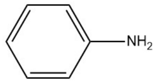
P173	3186.4	1.7	0.38	-0.14	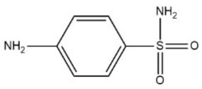
P289	2785.13	10.3	0.89	-0.14	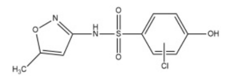
P305	1564.28	10.21	0.82	-0.35	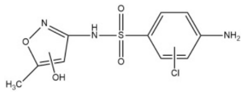
P195	1817.29	N/A	0.4	0.4	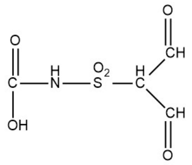
P110	330.3	6.17	0.59	0.19	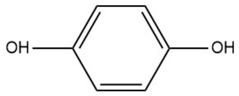

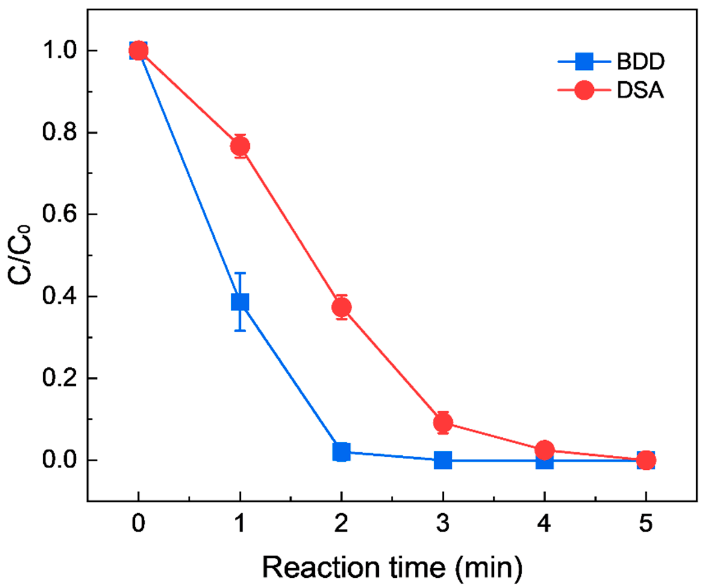

Fig. S1. Comparison of the efficiency of BDD and DSA electrodes for the electrochemical degradation of SMX. Reaction condition: current density = 4.44 mA/cm^2, [SMX] = 8 μmol/L, [NaCl] = 20 mmol/L, pH = 7.5±0.1.

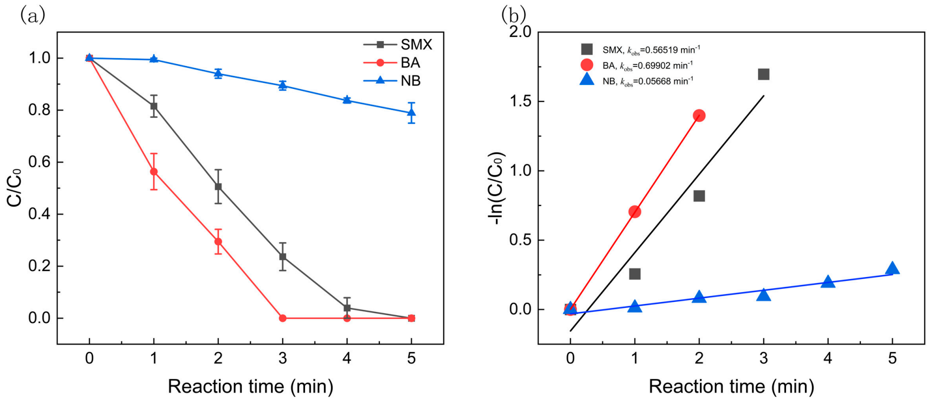

Fig. S2. The degradation of SMX, BA and NB in the competitive system. Reaction condition: current density = 4.44 mA/cm^2, [SMX] = [BA] = [NB] = 8 μmol/L, pH = 7.5±0.1, [NaCl] = 20 mmol/L.

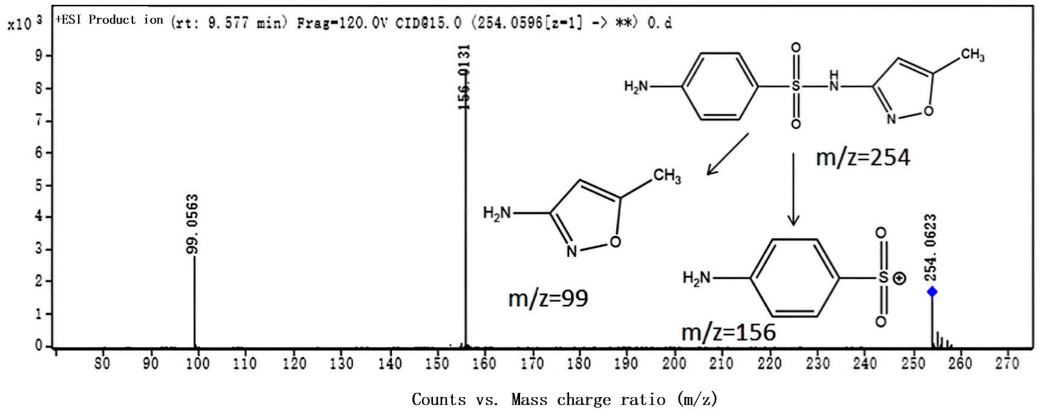

Fig. S3. Identification of SMX (m/z +254) and its fragment ions 156 and 99 ($[M+H]^+$).

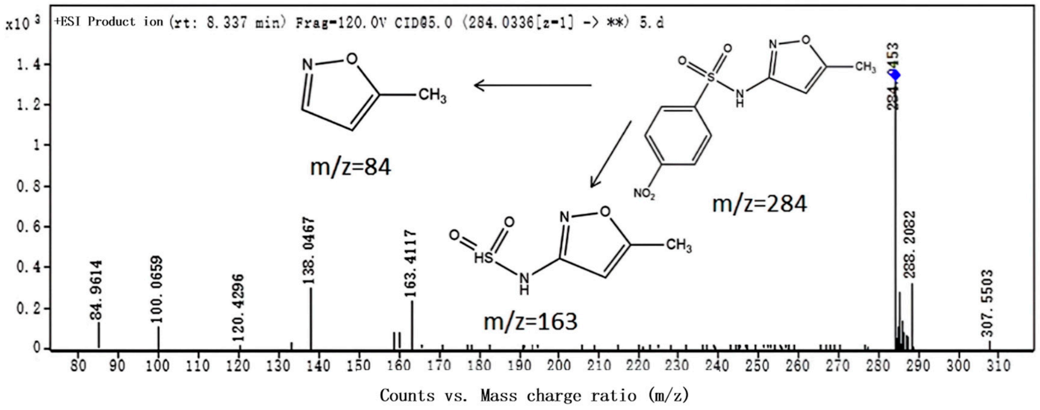

Fig. S4. Identification of P284 (m/z +284) and its fragment ions 84 and 163 ($[M+H]^+$).

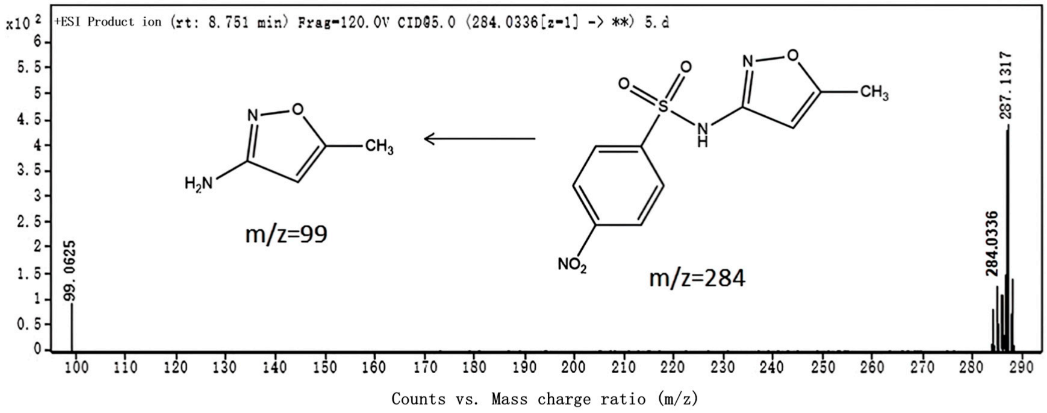

Fig. S5. Identification of P284 (m/z +284) and its fragment ions 99 ($[M+H]^+$).

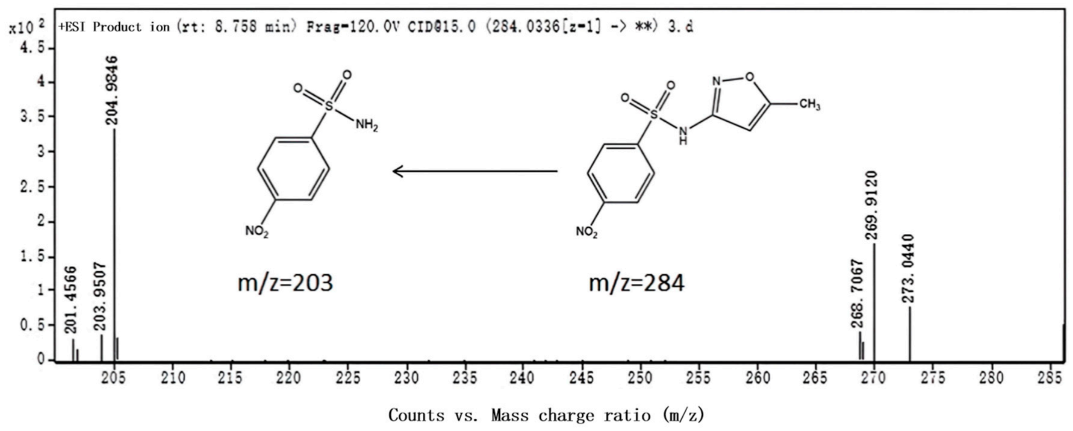

Fig. S6. Identification of P284 (m/z +284) and its fragment ions 203 ($[M+H]^{+}$).

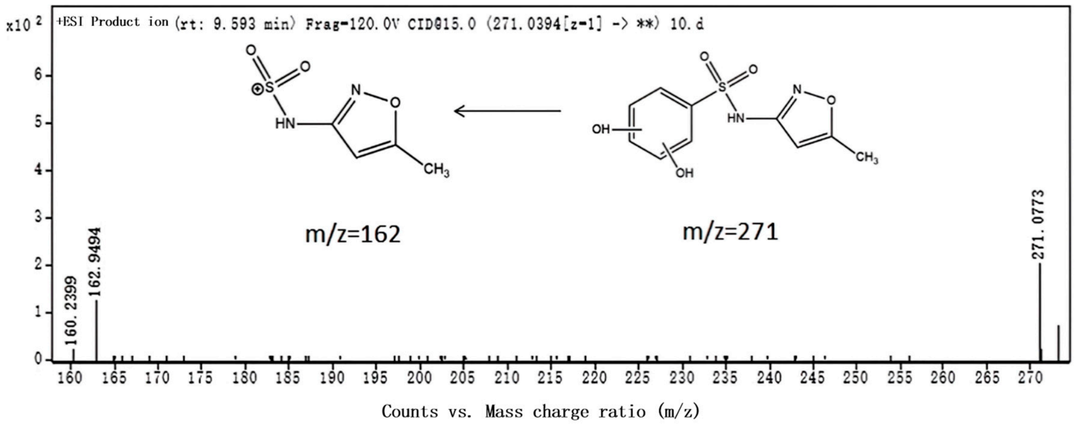

Fig. S7. Identification of P271 (m/z +271) and its fragment ions 162 ($[M+H]^{+}$).

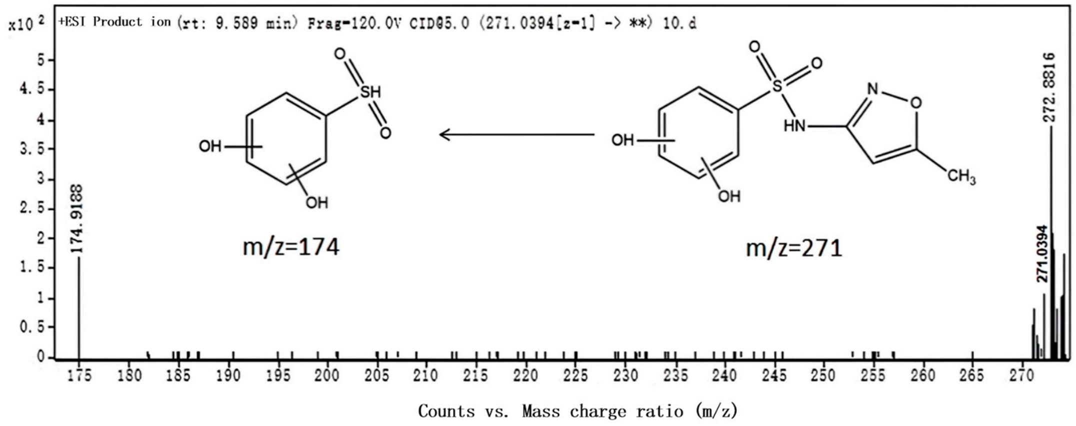

Fig. S8. Identification of P271 (m/z +271) and its fragment ions 174 ($[M+H]^{+}$).

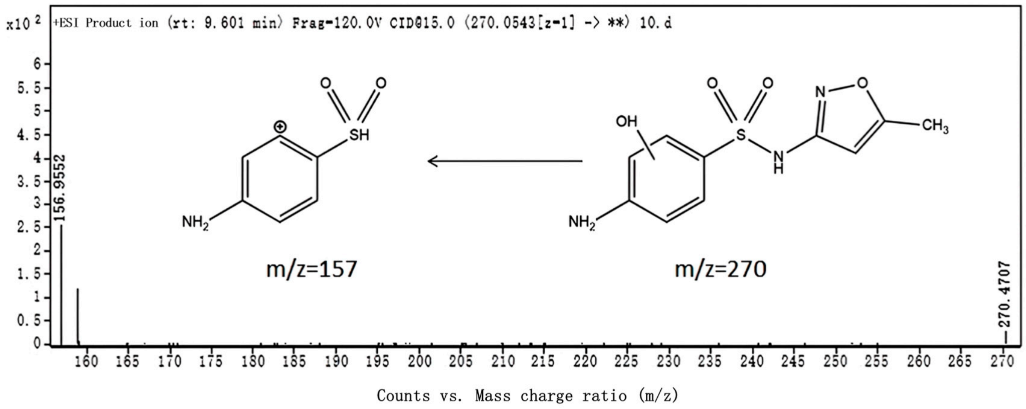

Fig. S9. Identification of P270 (m/z +270) and its fragment ions 157 ([M+H]$^+$).

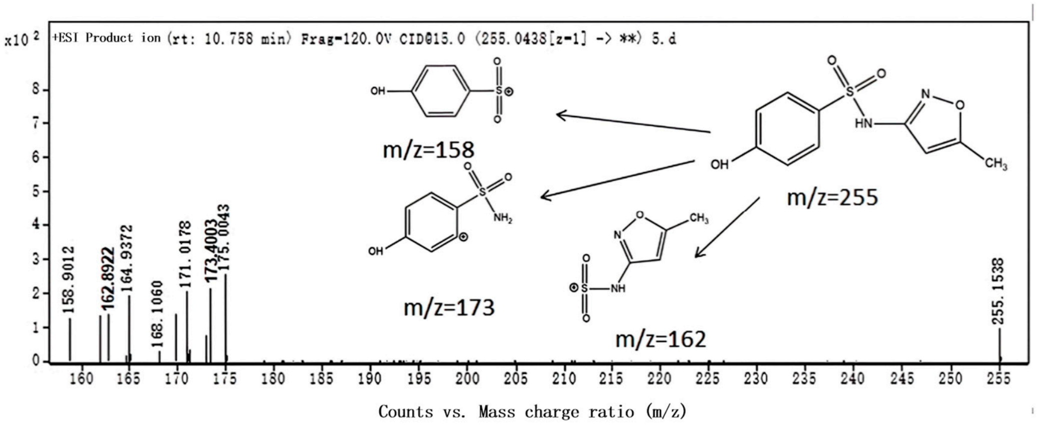

Fig. S10. Identification of P255 (m/z +255) and its fragment ions 158, 173 and 162 ([M+H]$^+$).

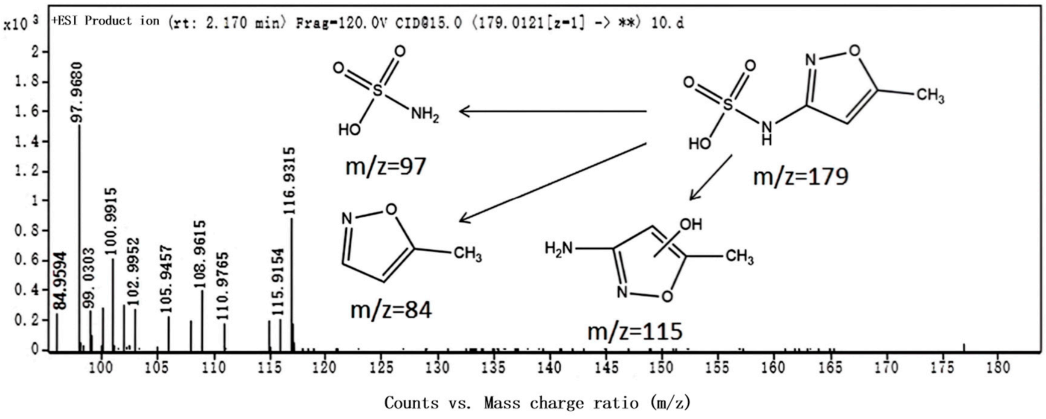

Fig. S11. Identification of P179 (m/z +179) and its fragment ions 97, 84 and 115 ([M+H]$^+$).

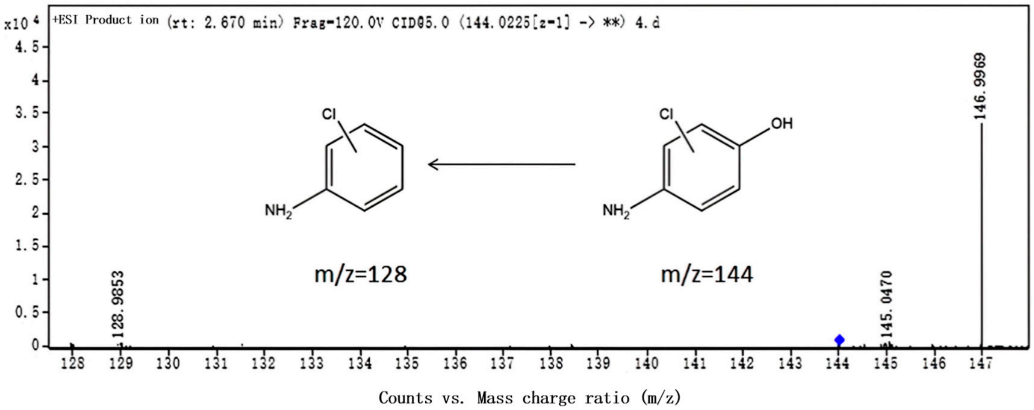

Fig. S12. Identification of P99 (m/z +99) and its fragment ions 85 and 72 ([M+H]$^+$).

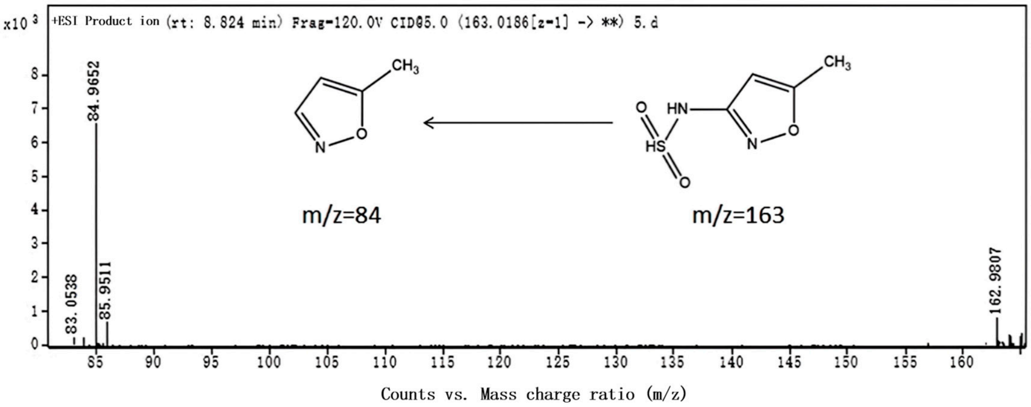

Fig. S13. Identification of P163 (m/z +163) and its fragment ions 84 ([M+H]$^+$).

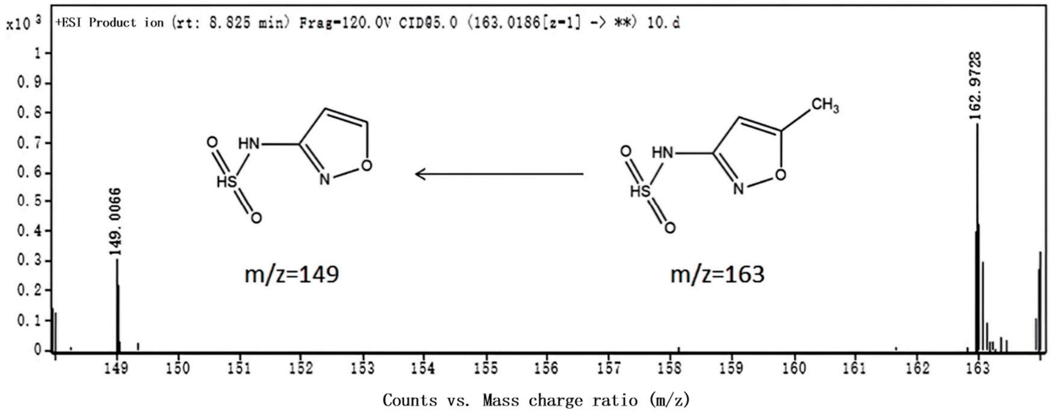

Fig. S14. Identification of P163 (m/z +163) and its fragment ions 149 ([M+H]$^+$).

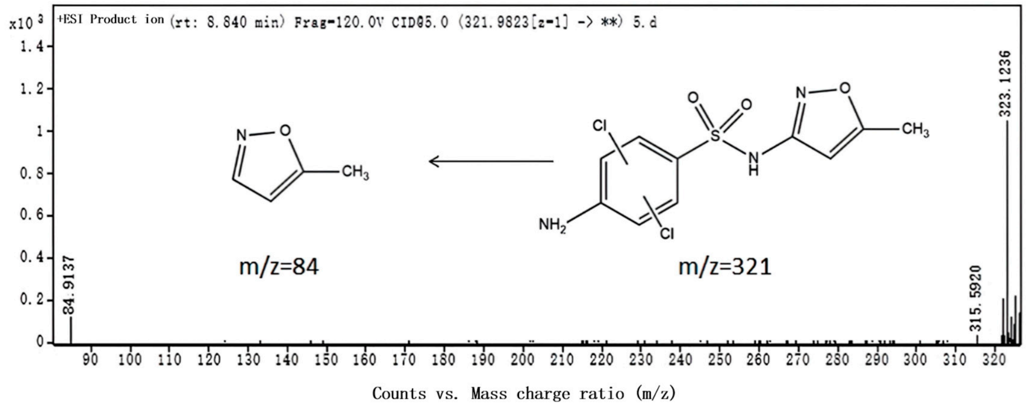

Fig. S15. Identification of P321 (m/z +321) and its fragment ions 84 ([M+H]$^+$).

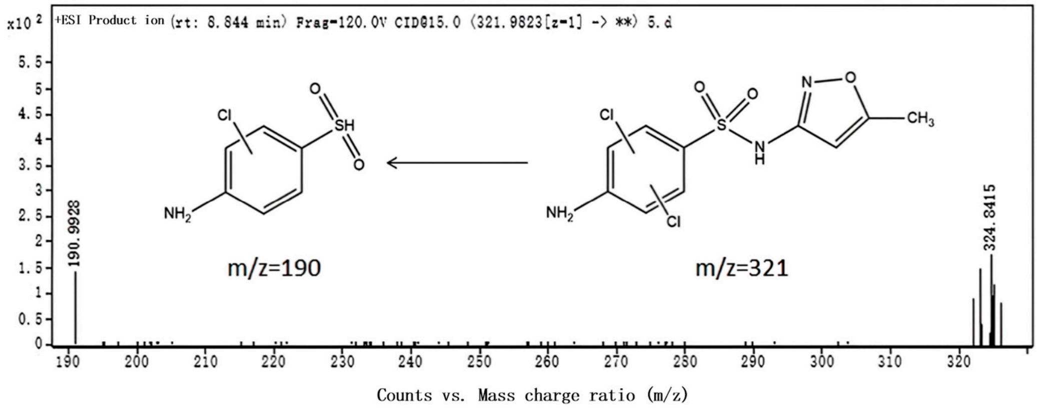

Fig. S16. Identification of P321 (m/z +321) and its fragment ions 190 ([M+H]$^+$).

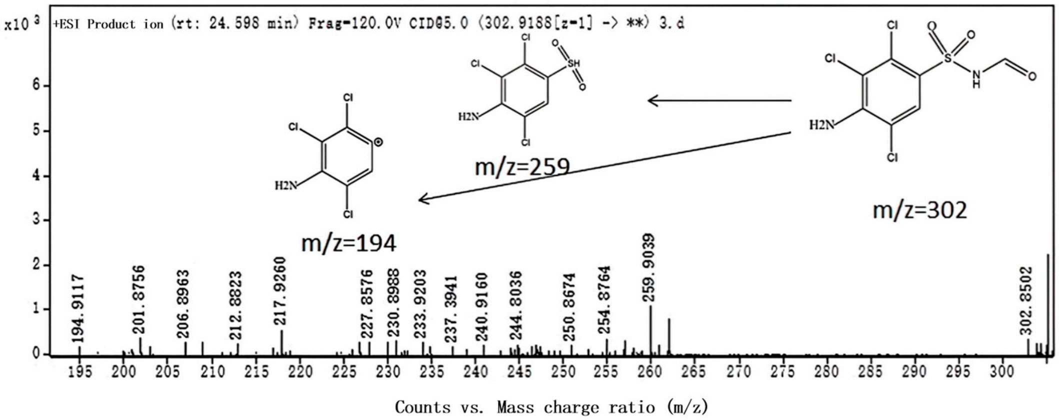

Fig. S17. Identification of P302 (m/z +302) and its fragment ions 259 and 194 ([M+H]$^+$).

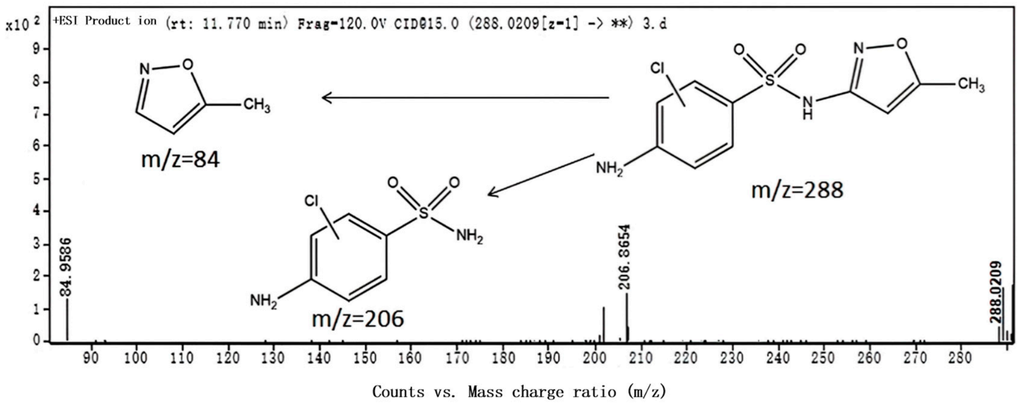

Fig. S18. Identification of P288 (m/z +288) and its fragment ions 84 and 206 ($[M+H]^+$).

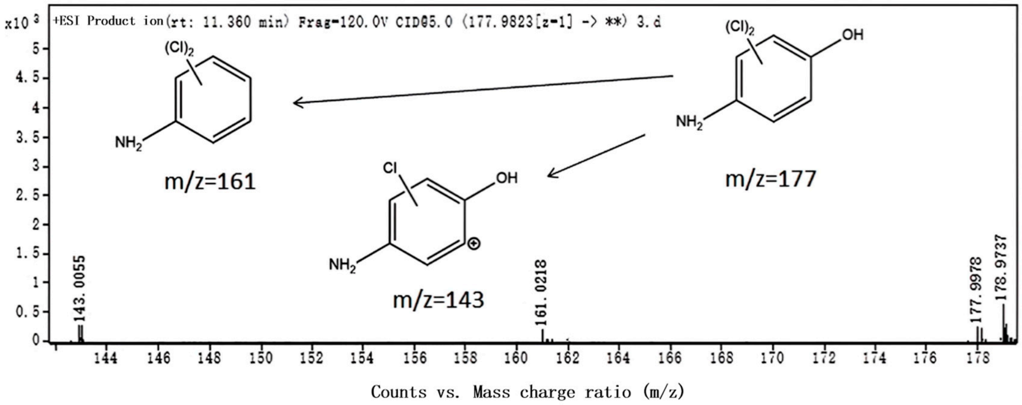

Fig. S19. Identification of P177 (m/z +177) and its fragment ions 143 and 161 ($[M+H]^+$).

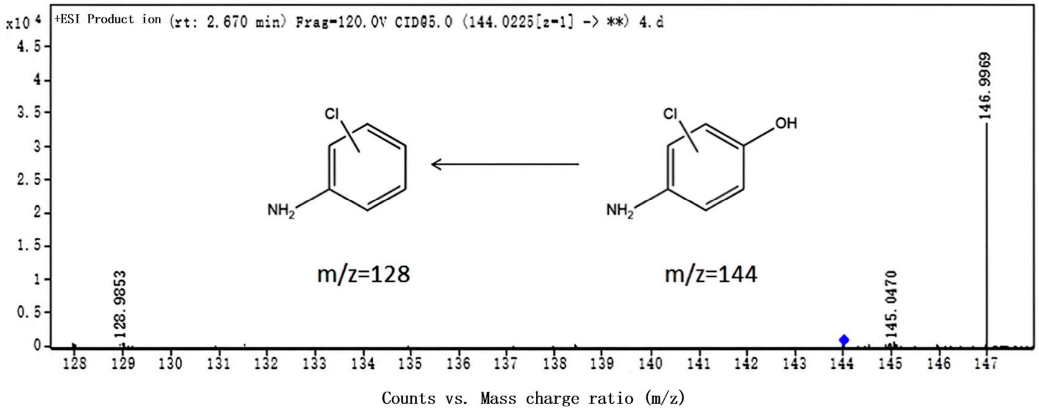

Fig. S20. Identification of P144 (m/z +144) and its fragment ions 128 ($[M+H]^+$).

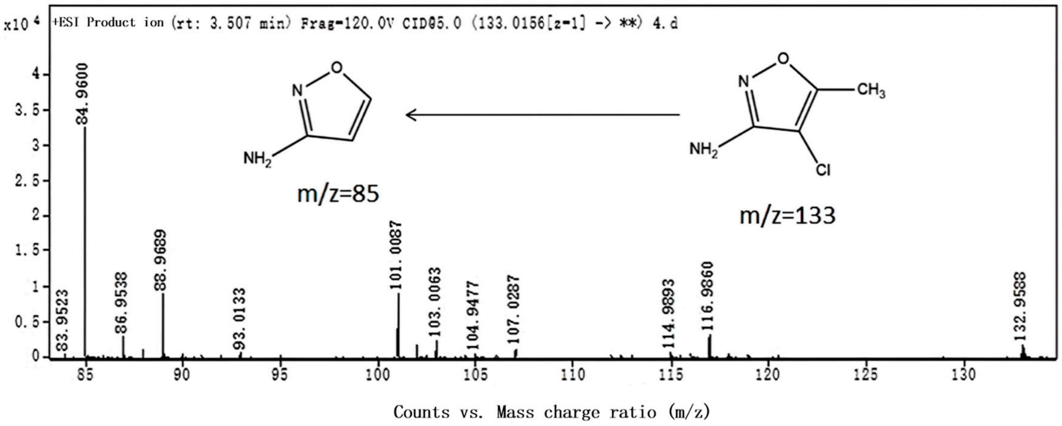

Fig. S21. Identification of P133 (m/z +133) and its fragment ions 85 ($[M+H]^+$).

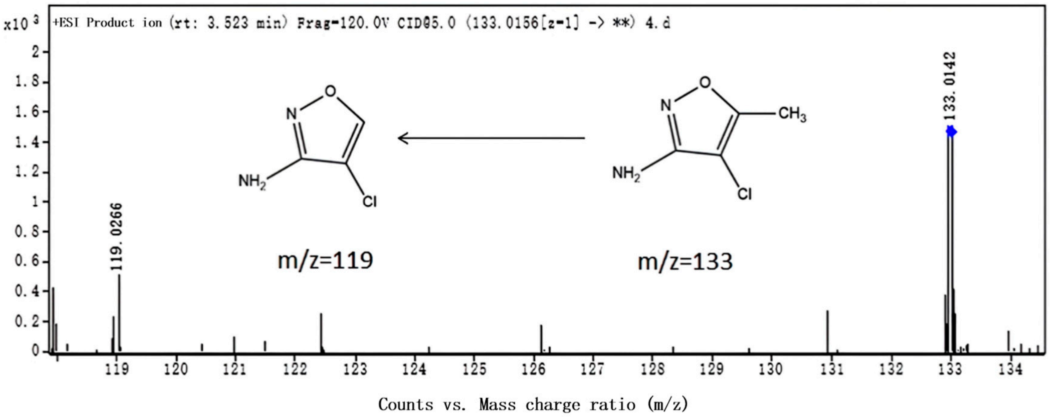

Fig. S22. Identification of P133 (m/z +133) and its fragment ions 119 ($[M+H]^+$).

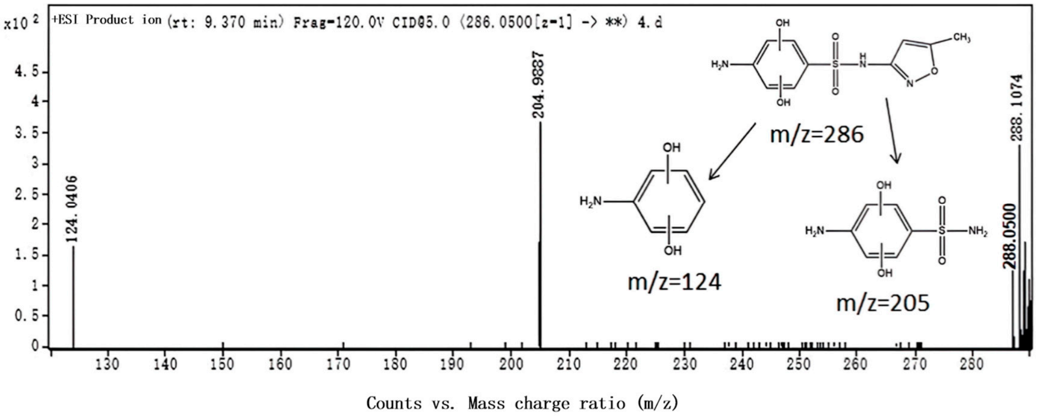

Fig. S23. Identification of P286 (m/z +286) and its fragment ions 124 and 205 ($[M+H]^+$).

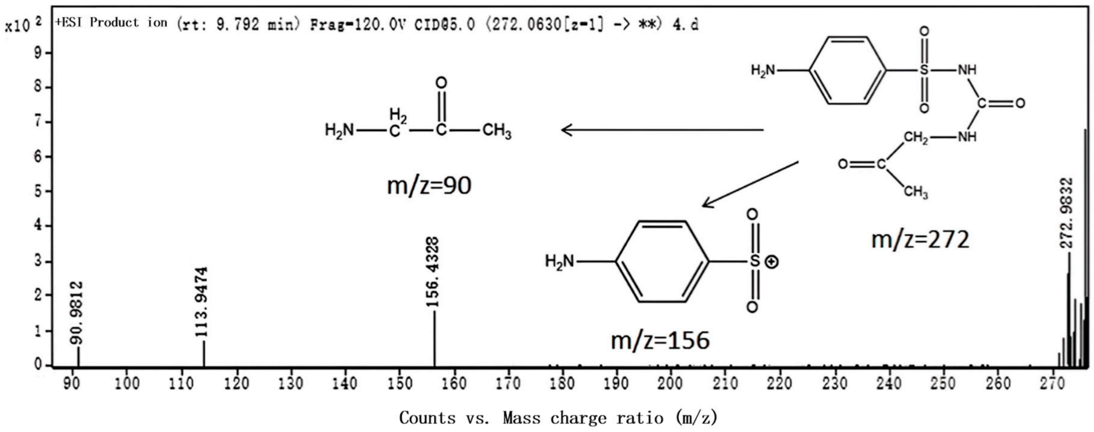

Fig. S24. Identification of P272 (m/z +272) and its fragment ions 90 and 156 ($[M+H]^+$).

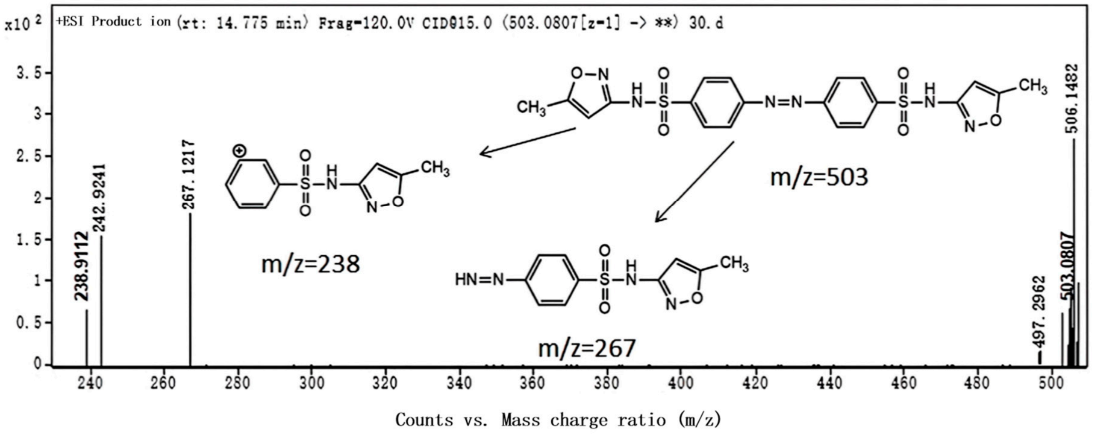

Fig. S25. Identification of P503 (m/z +503) and its fragment ions 238 and 267 ($[M+H]^+$).

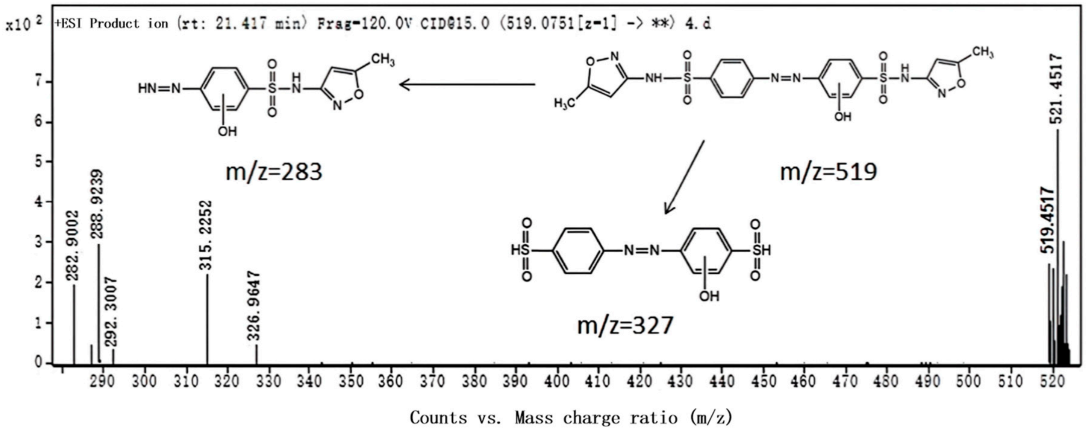

Fig. S26. Identification of P519 (m/z +519) and its fragment ions 283 and 327 ($[M+H]^+$).

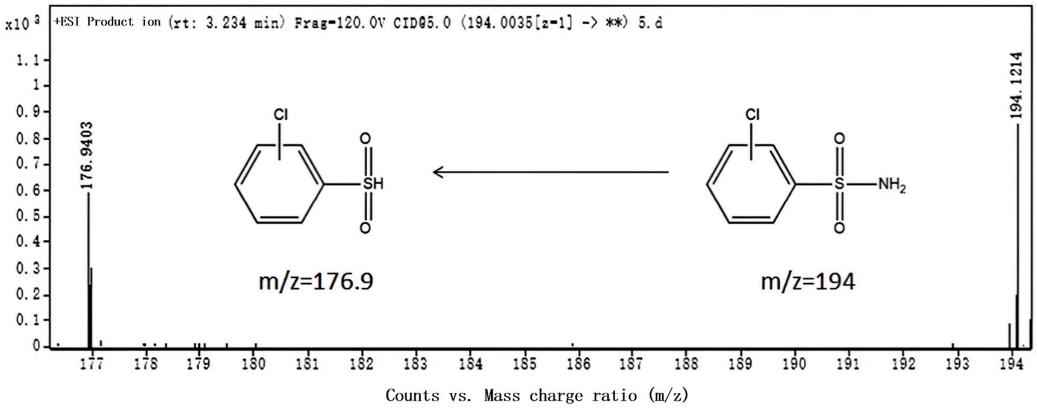

Fig. S27. Identification of P194 (m/z +194) and its fragment ions 176.9 ($[M+H]^+$).

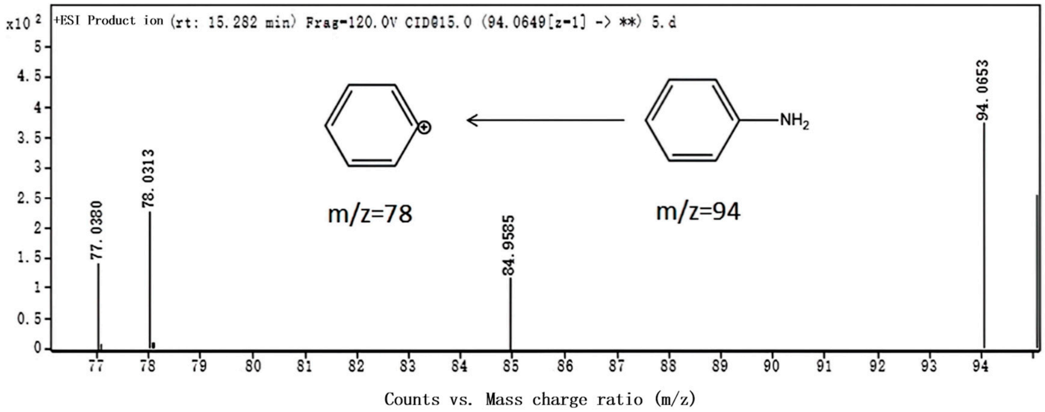

Fig. S28. Identification of P94 (m/z +94) and its fragment ions 78 ($[M+H]^+$).

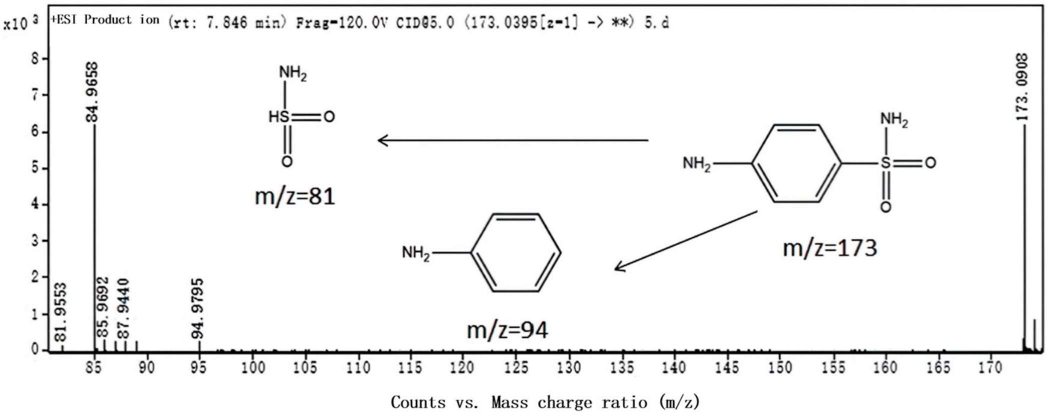

Fig. S29. Identification of P173 (m/z +173) and its fragment ions 81 and 94 ($[M+H]^+$).

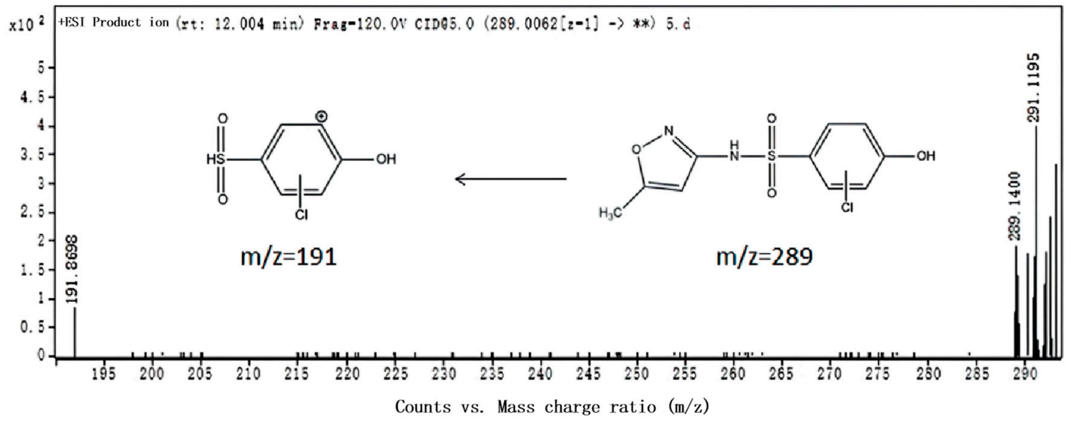

Fig. S30. Identification of P289 (m/z +289) and its fragment ions 191 ([M+H]$^+$).

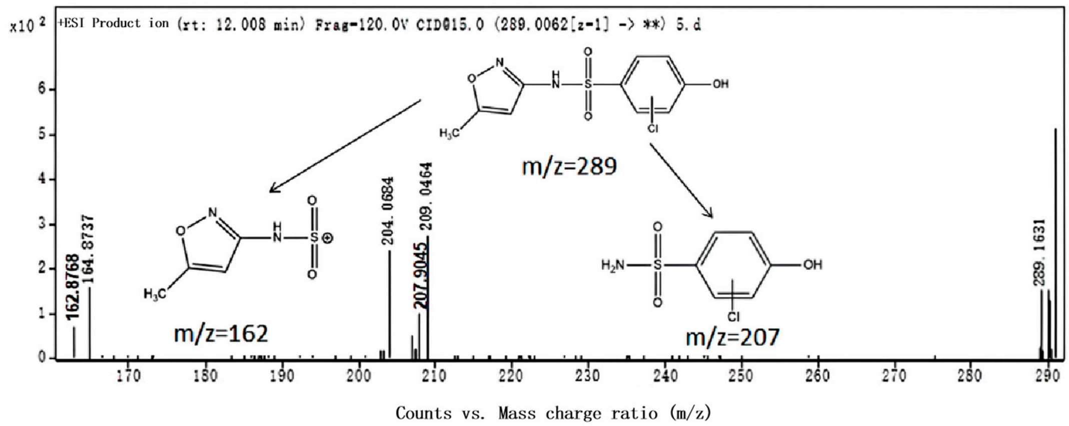

Fig. S31. Identification of P289 (m/z +289) and its fragment ions 162 and 207 ([M+H]$^+$).

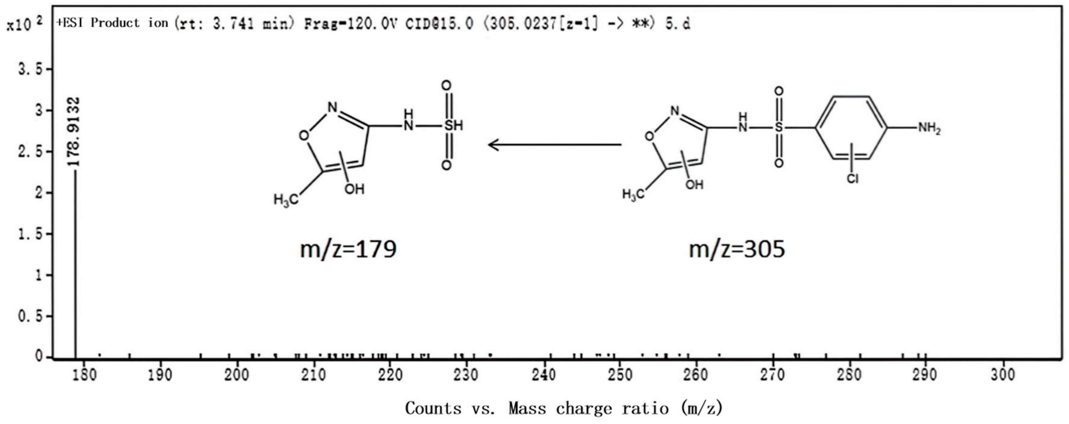

Fig. S32. Identification of P305 (m/z +305) and its fragment ions 179 ([M+H]$^+$).

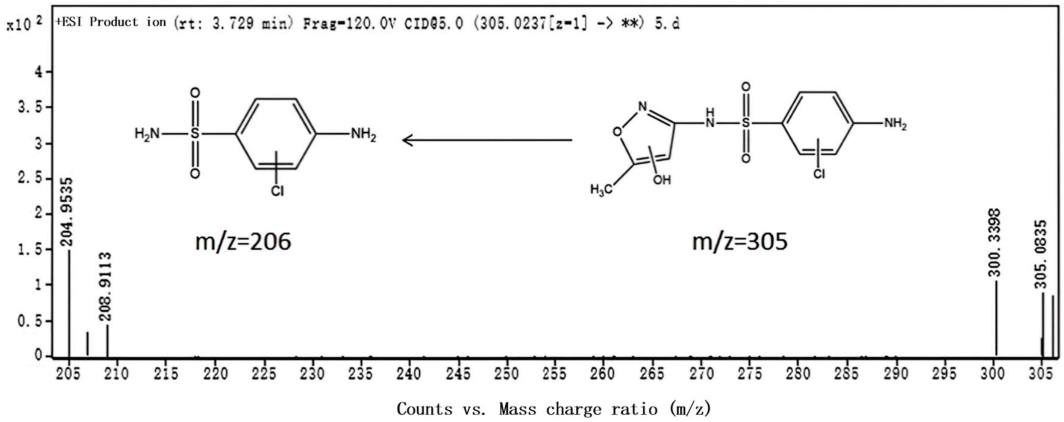

Fig. S33. Identification of P305 (m/z +305) and its fragment ions 206 ($[M+H]^+$).

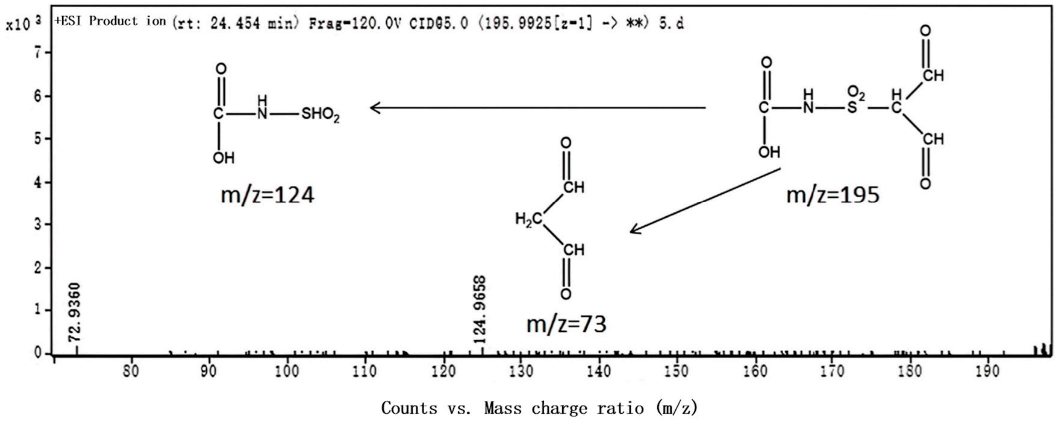

Fig. S34. Identification of P195 (m/z +195) and its fragment ions 124 and 73 ($[M+H]^+$).

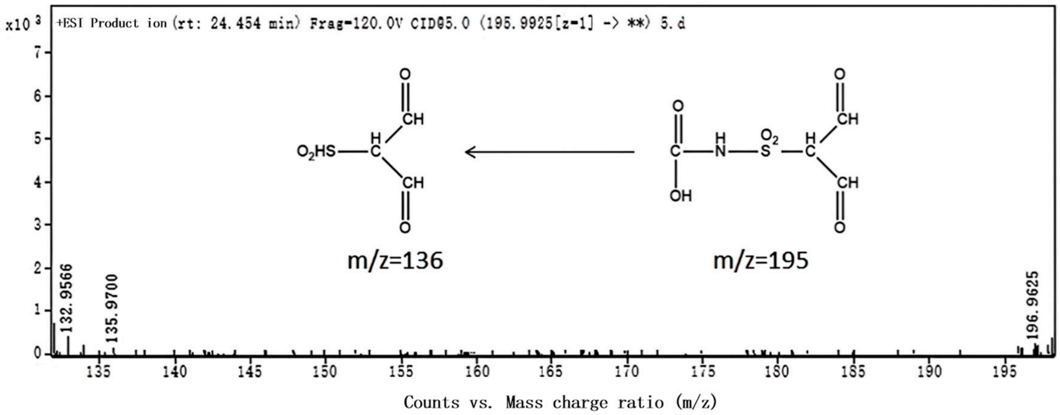

Fig. S35. Identification of P195 (m/z +195) and its fragment ions 136 ($[M+H]^+$).

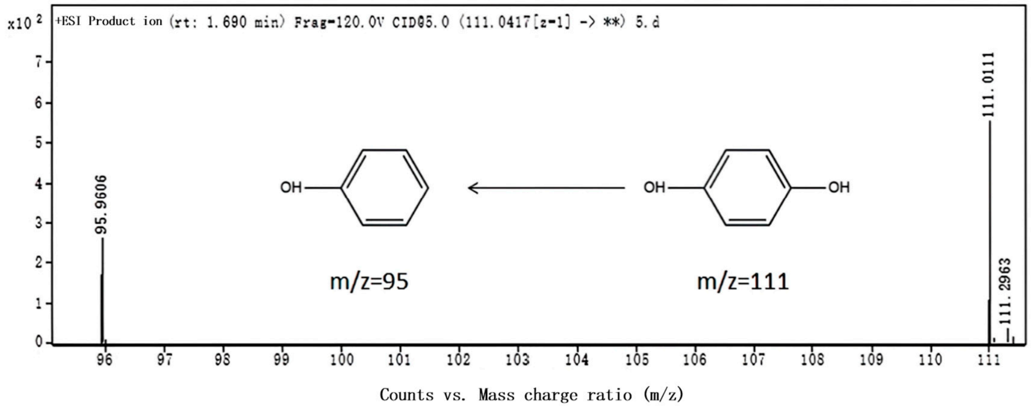

Fig. S36. Identification of P111 (m/z +111) and its fragment ions 95 ($[M+H]^+$).

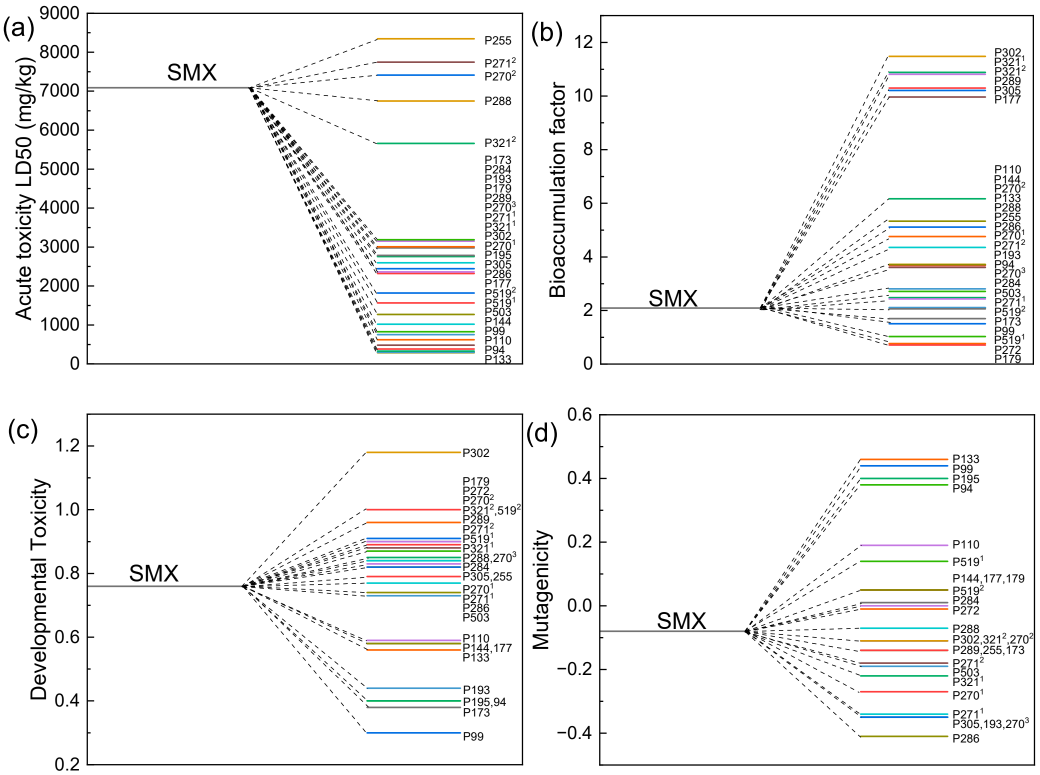

Fig. S37. (a) Acute toxicity, (b) bioaccumulation factor, (c) developmental toxicity and (d) mutagenicity. Reaction condition: current density = 4.44 mA/cm^2, [SMX] = 8 μmol/L, pH = 7.5 ±0.1, [NaCl] = 20 mmol/L.
